# Analysis of host-pathogen gene association networks reveals patient-specific response to streptococcal and polymicrobial necrotising soft tissue infections

**DOI:** 10.1186/s12916-022-02355-8

**Published:** 2022-05-04

**Authors:** Sanjeevan Jahagirdar, Lorna Morris, Nirupama Benis, Oddvar Oppegaard, Mattias Svenson, Ole Hyldegaard, Steinar Skrede, Anna Norrby-Teglund, INFECT Study group, Trond Bruun, Eivind Rath, Torbjørn Nedrebø, Per Arnell, Anders Rosen, Morten Hedetoft, Martin B. Madsen, Mattias Svensson, Johanna Snäll, Ylva Karlsson, Michael Nekludov, Vitor A. P. Martins dos Santos, Edoardo Saccenti

**Affiliations:** 1 Laboratory of Systems and Synthetic Biology, Wageningen University & Research, Stippeneng 4, 6708 WE Wageningen, the Netherlands; 2 Lifeglimmer GmbH, Markelstraße 38, 12163 Berlin, Germany; 3 Present affiliation: Department of Medical Informatics, Amsterdam Public Health Research Institute, Amsterdam UMC, University of Amsterdam, Meibergdreef 9, Amsterdam, Netherlands; 4 Department of Medicine, Division for infectious diseases, Haukeland University Hospital, Bergen, Norway; 5 Center for Infectious Medicine, Department of Medicine, Karolinska Institutet, Karolinska University Hospital, Huddinge, Sweden; 6 Department of Anesthesia, Centre of Head and Orthopaedics, Copenhagen University Hospital, Rigshospitalet, Copenhagen, Denmark; 7 Department of Clinical Medicine, University of Copenhagen, Copenhagen, Denmark; 8 Department of Clinical Science, University of Bergen, Bergen, Norway

**Keywords:** Bacterial infection, Dual RNA-seq, Polymicrobial infection, Single-sample networks, Streptococcus pyogenes, Transcriptomics

## Abstract

**Background:**

Necrotising soft tissue infections (NSTIs) are rapidly progressing bacterial infections usually caused by either several pathogens in unison (polymicrobial infections) or *Streptococcus pyogenes* (mono-microbial infection). These infections are rare and are associated with high mortality rates. However, the underlying pathogenic mechanisms in this heterogeneous group remain elusive.

**Methods:**

In this study, we built interactomes at both the population and individual levels consisting of host-pathogen interactions inferred from dual RNA-Seq gene transcriptomic profiles of the biopsies from NSTI patients.

**Results:**

NSTI type-specific responses in the host were uncovered. The *S. pyogenes* mono-microbial subnetwork was enriched with host genes annotated with involved in cytokine production and regulation of response to stress. The polymicrobial network consisted of several significant associations between different species (*S. pyogenes*, *Porphyromonas asaccharolytica* and *Escherichia coli*) and host genes. The host genes associated with *S. pyogenes* in this subnetwork were characterised by cellular response to cytokines. We further found several virulence factors including hyaluronan synthase, Sic1, Isp, SagF, SagG, ScfAB-operon, Fba and genes upstream and downstream of EndoS along with bacterial housekeeping genes interacting with the human stress and immune response in various subnetworks between host and pathogen.

**Conclusions:**

At the population level, we found aetiology-dependent responses showing the potential modes of entry and immune evasion strategies employed by *S. pyogenes*, congruent with general cellular processes such as differentiation and proliferation. After stratifying the patients based on the subject-specific networks to study the patient-specific response, we observed different patient groups with different collagens, cytoskeleton and actin monomers in association with virulence factors, immunogenic proteins and housekeeping genes which we utilised to postulate differing modes of entry and immune evasion for different bacteria in relationship to the patients’ phenotype.

**Supplementary Information:**

The online version contains supplementary material available at 10.1186/s12916-022-02355-8.

## Background

Necrotising soft tissue infections (NSTI) are devastating bacterial infections characterised by impairment and injury in any layer of the soft tissue compartment, extending from the epidermis to the deep musculature [1, 2]. These infections are relatively rare (0.2 to 15.5 per 100,000 people/year) but their aggressive nature poses severe threats due to the high risk of mortality and long-term disability which often results from the extensive tissue loss and amputations often prescribed to control the infections [3] due to the fact that progression is rapid, and early diagnosis is vital for improving the prognosis of affected patients [4–7].

NSTIs can be caused by either a single bacterial species (monomicrobial NSTI, or Type 2 NSTI) or by multiple species (polymicrobial NSTI, or Type 1 NSTI), and the relative occurrence of the two types of NSTI differs significantly based on the geography and patient characteristics [3, 8].

*Streptococcus pyogenes* is the most common pathogen in monomicrobial NSTIs [3], but other streptococcal species [9] and *Staphylococcus aureus* are also known to cause monomicrobial NSTIs [10]. In this study, we only focus on type 2 NSTI caused by *S. pyogenes*, as well as polymicrobial NSTIs that are associated with a mixture of obligate anaerobic and facultative anaerobic bacteria [11], such as *Enterobacteriaceae*, *Bacteroides spp.*, *Porphyromonas spp.*, *Prevotella spp.*, *Peptostreptococcus spp.* and *Clostridium spp.* [1, 12]

Monomicrobial NSTIs caused by *S. pyogenes* have been studied extensively and many of the virulence factors and toxins expressed by the bacterium to colonise the host tissue and bypass the host immune defences have been characterised [13]. In contrast, the pathogenic strategies and the complex dynamics of bacterial communities underlying polymicrobial NSTIs are poorly understood. One of the major limitations in our understanding of NSTI at the molecular level (and of bacterial infections in general) is the insufficient information about the web of molecular interaction, also known as interactome, between pathogens and the human host. In contrast with Mendelian diseases, where one or few genes can be directly linked to the disease, bacterial infections arise from the complex interactions between bacteria, the host immune system, predisposition, risk and environmental factors [14].

Interactomics focuses on the representation and the analysis of the interactions between biological features on a global scale [15] using network approaches to simplify a complex system like, in this case, a bacteria-host system, and to summarise it as components (nodes) and interactions (edges) between them [16]. Both nodes and edges can be different in nature, depending on the type of interactome considered. In this study, nodes are human and bacterial genes and edges represent the existence of a correlation between the expression profiles of these genes, thus representing the mutual response of host and pathogen and providing a global view of the observed interactions at the molecular level [17].

The present study builds and expands (on) the data obtained in the largest cohort study of NSTI patients in the world to date, the INFECT study. Thänert et al. [18] analysed dual RNA sequencing of NSTI patient biopsies together with microbial community profiling using 16S rRNA sequencing data [18] collected within the INFECT study [19], and showed that gene expression profiles of tissues from NSTI patients differed significantly between monomicrobial streptococcal and polymicrobial infections, identifying the core inflammatory signatures in both instances.

Here, we used network analysis to explore relationships between co-expressed host and bacterial gene pairs, complementing and expanding the results of Thänert et al. [18] by considering a larger number of samples with the aim to provide insight into the interaction and dynamics between the pathogens and the host and illuminate some of the underlying molecular mechanisms in the pathophysiology of NSTI using a systems biology approach [15, 17].

## Methods

### Study design

The study is founded in the INFECT study on clinical and pathogenesis in NSTI, where patients were included by prospective enrolment through 4.5 years in five Scandinavian referral hospitals (see Table 1). Study design and presentation of clinical results are detailed elsewhere [6, 19, 20]. The INFECT study is registered at ClinicalTrials.gov (NCT01790698).

Tissue biopsies and plasma samples on the day of hospital admission (day 0) were obtained from patients diagnosed with NSTI and admitted to Karolinska University Hospital in Stockholm, Copenhagen University hospital Rigshospitalet, Blekinge County Council Hospital in Karlskrona, Sahlgrenska University Hospital in Gothenburg and Haukeland University Hospital in Bergen in the framework of the EU project INFECT (https://permedinfect.com/projects/peraid/).

Diagnosis of NSTI was based on the presence of necrotic or deliquescent soft tissue with widespread undermining of the surrounding tissue. Patients were excluded in absence of reports of necrotic or deliquescent tissue. More details on patient characteristics and study design can be found in [19].

### Ethical considerations

The INFECT study was conducted in accordance with the Declaration of Helsinki and was approved by the regional Ethical Review Board at the Karolinska Institutet in Stockholm, Sweden (Ethics Permits: 2012/2110-31/2), the National Committee on Health Research Ethics in Copenhagen, Denmark (Ethics permits: 1151739), the regional Ethical Review Board in Gothenburg, Sweden (Ethics permits: 930-12) and Bergen, Norway (2012/2227/REC West). All experiments were performed in accordance with the approved ethics applications specified above. All patients provided written informed consent. The INFECT study is registered at ClinicalTrials.gov (NCT01790698).

### Experimental methods

#### RNA-seq sample preparation and sequencing

This study makes use of RNA-seq samples used in [18, 19] plus additional data not available at the time. All samples were handled and processed as described in [18, 19].

#### Sample selection

**Fig. 1:**
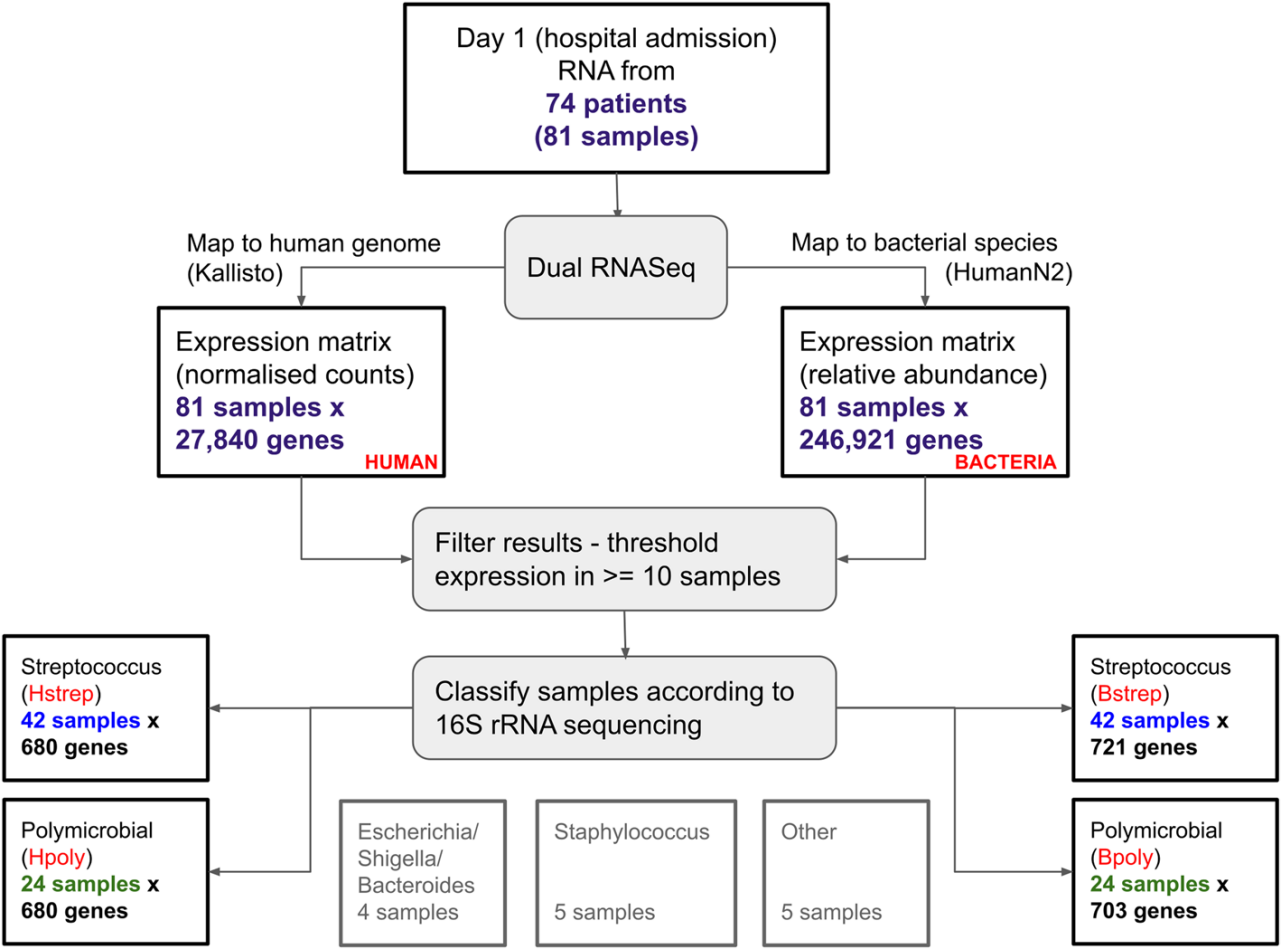
Flowchart for sample selection, mapping and filtering of dual RNA-seq for the generation of gene expression matrices for *S. pyogenes* monomicrobial infections (Hstrep -Human, Bstrep -Bacteria) and polymicrobial infections (Hpoly – Human, Bpoly -Bacteria). Bacterial and Human gene expression are measured on the same tissue biopsies from NSTI patients. Clinical information associated with patients and samples\biopsies is given in Table 1

In line with the study by Thänert et al., we retained all those subjects/samples for which dual RNA-seq data (i.e., transcriptomics data for both host and pathogen) was available together with 16S bacterial rRNA gene sequencing data collected on the day of hospital admission: this results in 81 samples available for analysis (Fig. 1).

We followed the classification established in [18] that assigned the samples to 5 different types of infection based on their associated bacterial composition according to 16S rRNA gene sequencing. Classification was based on average-linkage hierarchical agglomerative clustering using the relative abundance of the identified bacterial communities. The optimal number of clusters in the resulting sample dendrogram was determined using the J-index, and distinct specimen clusters were defined to represent different types of NSTIs. This information was obtained from the Supplementary Data 3 from [18].

The sample size for *Staphylococcus*, *Escherichia*/*Shigella*/*Bacteroides* and other was not large enough to build robust correlation networks, thus we focused on the two larger groups, namely samples from patients with *S. pyogenes* and polymicrobial NSTI. We indicate with Hstrep and Hpoly the matrix of human gene expression measured on biopsies obtained from patients with diagnosis *S. pyogenes* NSTI and polymicrobial NSTI, respectively; we indicate with Bstrep and Bpoly the matrix of bacterial gene expression for *S. pyogenes* NSTI and polymicrobial NSTI, respectively. Relevant clinical information associated with the samples\biopsies can be found in Table 1

**Table 1:** Clinical parameters associated with the patients whose biopsies were analysed with dual RNA-seq and used to build the host-pathogen interactome in NSTI

	Monomicrobial NSTI	Polymicrobial NSTI
Age (years)	57 (44–61)	55 (46.5–64)
Sex		
Female/male (%)	12 (34.3%)/ 23 (65.7%)	9 (39.2%)/ 14 (60.8%)
Outcome		
Mortality 30 days (%)	1 (2.86%)	4 (17.4%)
Mortality 90 days (%)	3 (8.57%)	4 (17.4%)
Mortality 365 days (%)	6 (17.14%)	6 (26.1%)
Hospital		
Righospitalet Copenhagen	13 (15)/35 (42)	11 (11)/ 24 (25)
Karolinska University Hospital	7 (8)/35 (42)	8 (8)/ 24 (25)
Sahlgrenska University Hospital	5 (6)/35 (42)	2 (2)/ 24 (25)
University of Bergen	10 (13) /35 (42)	2 (3)/ 24 (25)
Laboratory values		
Haemoglobin (g/dl)	10.5 (2.51–11.76)	8.8 (7.9–10.55)
White blood cells (10^9/l)	13.4 (11.15–16.625)	12.3 (8.7–15.7)
C-reactive protein (mg/l)	207 (152–293.25)	293 (140–343)
Creatinine (μmol/l)	123 (87.75–233.75)	116.5 (81.25–180.75)
SOFA score	9 (5–11)	8 (6–11)
SAPS II	40 (35–51)	40.5 (29–48)
Location of infection in patients		
Head and neck (%)	7 (20%)	9 (39.1%)
Upper extremities including thoracic involvement (%)	14 (40%)	3 (13%)
Abdomen and ano-genital area (%)	5 (14.3%)	12 (52.2%)
Lower extremities (%)	15 (42.9%)	5 (21.7%)

Median and Interquartile ranges (Lower Quartile – Upper Quartile) are given. Under the section “Hospital”, the number of patients admitted to and the number of biopsies taken (in brackets) from the hospitals are shown. The number of patients may not coincide with the number of biopsies since multiple biopsies can be taken from the same patient. For instance, “13 (15)/35 (42)” means that 13 out of the 35 patients and 15 out of the 42 biopsies were from Righospitalet; since patients can have infection in multiple locations, percentage may not add to 100%

### Bioinformatics analysis

RNA sequences were mapped against both human and bacterial genomes to obtain gene expression of both the host and resident pathogens. Quality control was performed with the tool FASTQC [21]. The mapping tool Kallisto [22] was used to map the sequences against the human genome (GRCh38 release 91). The same sequences were also mapped against several bacterial genomes using the published pipeline HUMAnN2 [23] from the UniRef database [24]. The final data sets for the *Streptococcus* monomicrobial classified samples contained 680 human genes and 721 bacterial Uniref90 sequences, and the polymicrobial classified samples contained 680 human genes and 703 bacterial Uniref90 sequences. Details of the methods used can be found in Additional file 1: Section S1.

### Gene-gene association network inference

Gene-Gene Association networks were built with an algorithm developed to be robust against variation in sample size and noise called the Probabilistic Context Likelihood of Relatedness on Correlation (PCLRC) algorithm [25, 26]. We used partial correlations obtained from a Gaussian Graphical Model (GMM, GeneNet R package implementation) as a measure of association\covariation between human and bacterial genes [27, 28]. A significant correlation *r*_*ij*_ between the *i*th host and *j*th pathogen gene was established if the corresponding Benjamini-Hochberg adjusted *P-*value < 0.05 [29].

### Functional analysis

TopGO R-package v2.42.0 was used for functional category enrichment analysis [30] using the human genes from each bacterial subnetwork as the target sets of interesting genes and the list of all genes from human genome build GRCh38.p12 downloaded from Ensembl Biomart [31] as the background set. The Biological Process ontology from Gene Ontology was used [32], and the Fisher’s exact test was selected to calculate statistical significance of enrichment for the genes of interest.

### Inference of host-pathogen gene association networks at the patient level

We used the Linear Interpolation to Obtain Network Estimates for Single Samples (LIONESS) method to infer the single-sample networks [33]. Estimation was done separately for streptococcal and polymicrobial NSTI as described in [34]. For sample *q* (containing gene expression profiles for host and pathogen from the same biopsy) out of *n* samples, the corresponding LIONESS single-sample network is obtained as described in Section 1.10 in Additional file 1. The networks have been estimated using the same approach described in Network Inference section using partial correlations.

### Clustering of patients based on single-sample networks

Each *m* × *m* single-sample network can be reduced to a ½*m*(*m*-1) × 1 vector containing the perturbations (edges) of the host-pathogen gene correlation. We collapsed these vectors in two matrices of size 2556 × 42 and 2691 × 24. For each matrix, pairwise distances (Euclidean) among samples were calculated, and hierarchical clustering was applied using the Ward linkage method [35].

Single-sample network edges were ranked for each group using the edge relevance as defined in Eq. 4 in Additional file 1: Section 3.12. For each group we retained the 10 most relevant associations.

## Results

A total of 66 samples were included, comprising 42 monomicrobial *Streptococcus pyogenes* NSTIs and 24 polymicrobial cases. The most abundant genera detected across all samples are *Streptococcus*, *Fusobacterium*, *Peptostreptococcus*, *Parvimonas*, *Peptoniphilus*, *Porphyromonas*, *Anaerococcus*, *Bacteroides* and *Escherichia*. We detected sequences unique to the following species: *Streptococcus pyogenes*, *Streptococcus dysgalactiae*, *Escherichia coli*, *Porphyromonas asaccharolytica*, *Parvimonas micra* and *Prevotella oris*. The monomicrobial streptococcal NSTI samples were restricted to patient cases with *Streptococcus pyogenes* infection. A principal component analysis plot of *S. pyogenes* and polymicrobial NSTI gene expressions and a Random Forest classifier discriminating between them can be found in Additional file 1: Fig. S1.

### Host-pathogen gene interaction networks

**Fig. 2:**
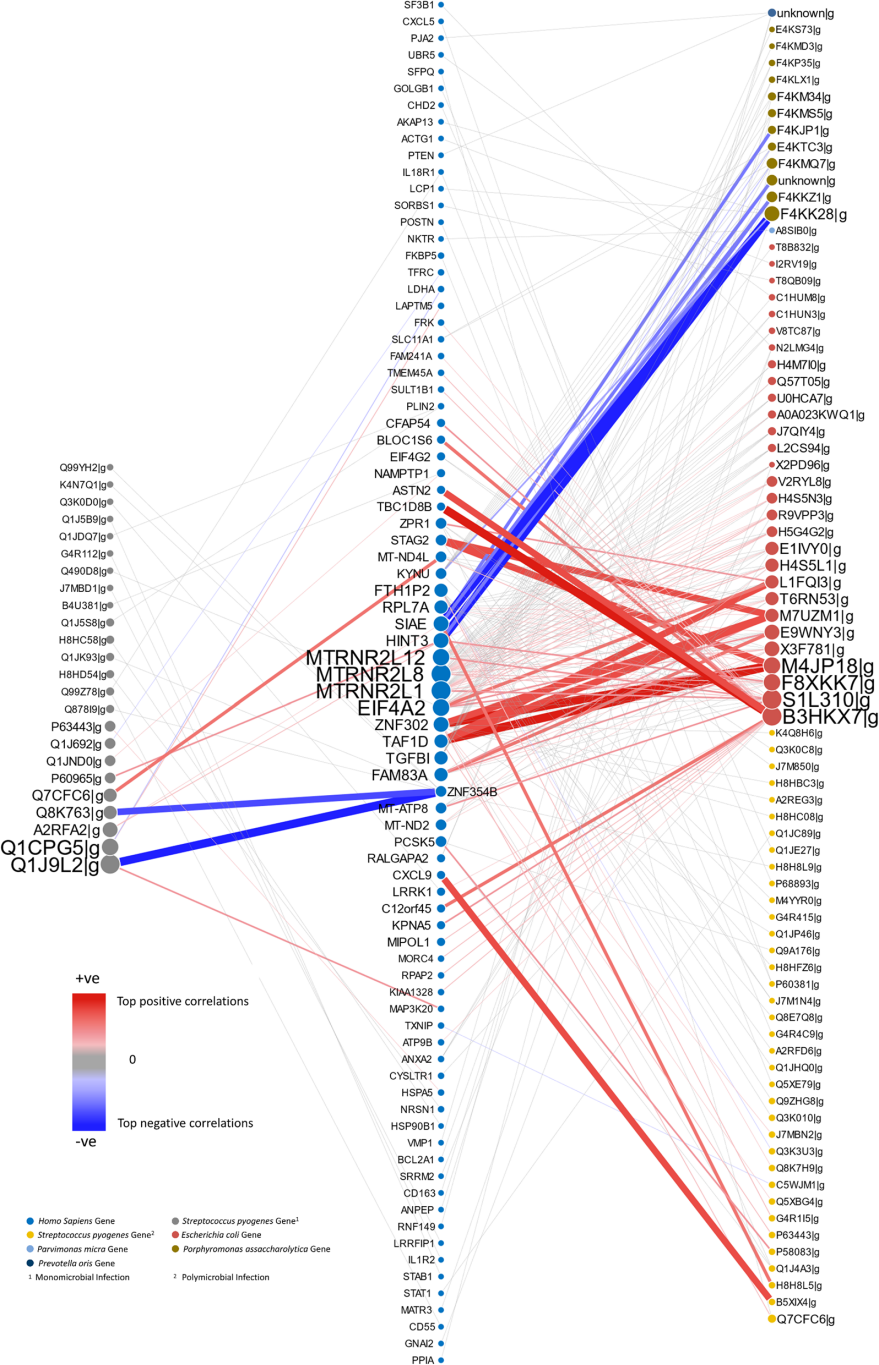
Interactome network of the host-pathogen gene expression profiles derived from Dual RNA-seq of tissue biopsies of NSTI patients. The central column contains human genes, the left column contains *S. pyogenes* genes (i.e. bacterial genes found to be associated with human genes in monomicrobial NSTI), the right column contains genes from several bacterial species (i.e. bacterial genes found to be associated with human genes in polymicrobial NSTI). Nodes are colour coded by bacterial species; the node size is proportional to the node degree (connectivity, i.e. the number of associated genes; see Additional File 1, Equation S3). Red edges indicate positive partial correlation; blue edges indicate negative partial correlation; the colour intensity and the edge width are proportional to the magnitude of the partial correlations

The interaction networks between human and bacterial genes are shown in Fig. 2. The network specific to monomicrobial *S. pyogenes* NSTI comprises the interaction of 20 human and 24 *Streptococcus pyogenes* genes, while the network for polymicrobial NSTI consists of 69 human and 79 bacterial genes.

We observed NSTI type-specific responses in the host, with different sets of human genes highly correlated with bacterial gene expression depending on whether the infection is caused by *S. pyogenes* or by multiple bacteria. We found the polymicrobial correlation network to be divided into subnetworks with genes from three bacterial species (*S. pyogenes*, *E.coli* and *P. asaccharolytica*) that have a high relative abundance over the samples. While the input gene expression matrix also contained several gene sequences for *Parvimonas micra* and *Prevotella oris*, only a single gene interaction with a host gene for each of these species was observed in the resulting networks.

**Fig. 3:**
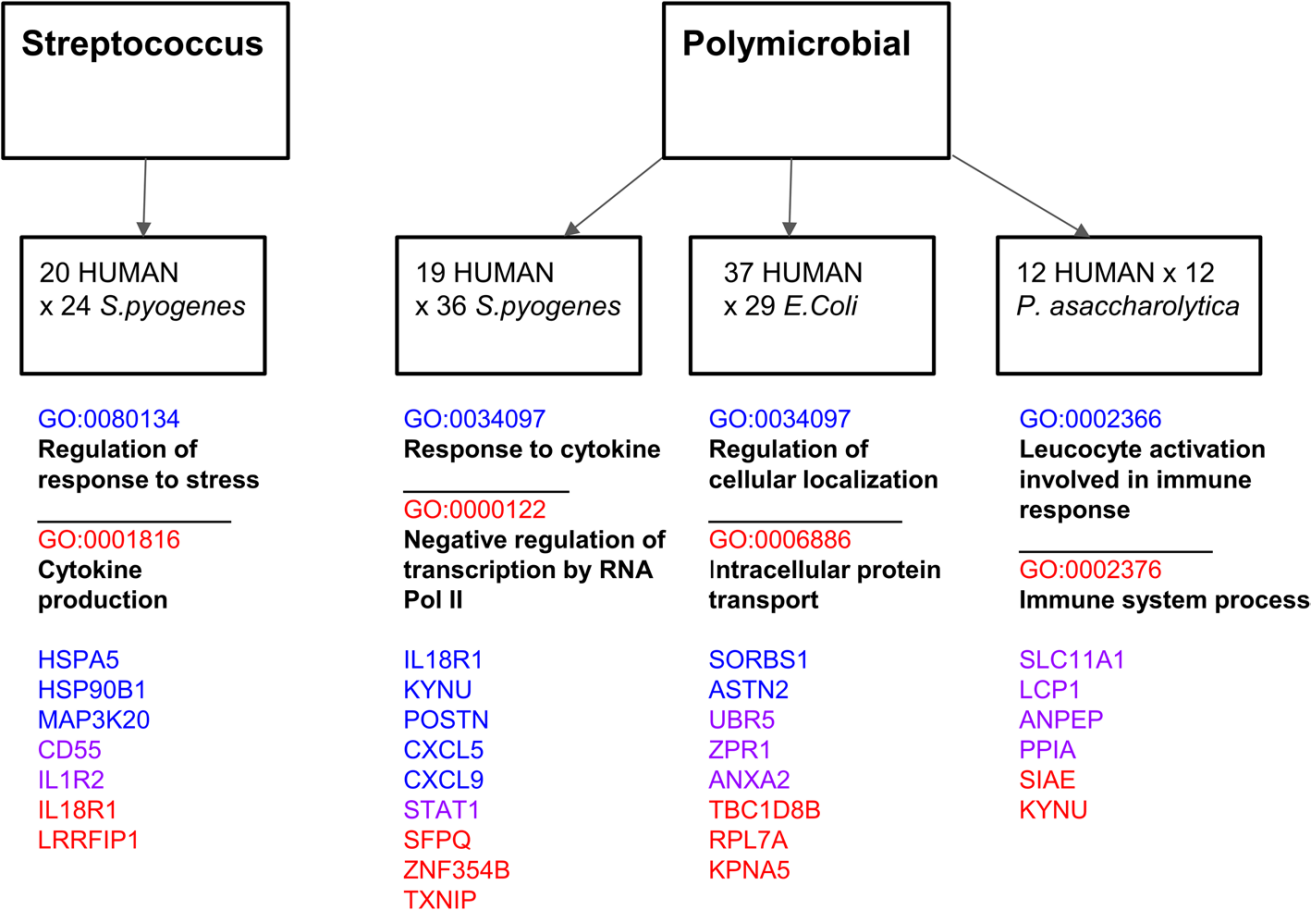
GO (Gene Ontology) enrichment analysis of the human and bacterial genes found to be associated and visualised in Fig. 2. Results are given for monomicrobial streptococcal (*S. pyogenes*) and for the polymicrobial NSTI. Gene names listed in blue indicate genes annotated by the GO term also indicated in blue, and gene names in red are annotated by the GO term in red. Genes given in purple colour are annotated by both GO terms. A different type of infection elicits different response patterns in the host

The genes from these association networks were isolated based on species for enrichment analysis. The set of human genes in the *S. pyogenes* monomicrobial network (consisting of *S. pyogenes* genes and associated human genes) were significantly enriched (adjusted *P*-value <0.05) in GO terms for cytokine production (GO:0080134) and regulation of response to stress (GO:0001816), which include genes coding for the interleukin receptors (IL1R2, IL18R1), CD55 and the heat shock proteins, HSPA5 and HSP90B1 (Fig. 3). The set of genes in the *S. pyogenes* subnetwork from the polymicrobial samples were significantly enriched in the GO term, cellular response to cytokine (GO:0034097), involving the genes for STAT1, IL18R1, POSTN, CXCL9, CXCL5, demonstrating different responses to mono- versus polymicrobial *S. pyogenes* NSTI.

We observed a strong negative association between the human gene Zinc-Finger Protein 354B (ZFN354B) and three streptococcal genes, *sic1* (Q1J9L2), SpyM3_0968 (Q8K763) and MGAS9429_Spy1542 (Q1JK93) in monomicrobial *S. pyogenes.* We also observed strong associations of *S. pyogenes* gene SpyM3_0408 (Q7CFC6) with TATA Box-binding protein-associated factor1D (TAF1D) in polymicrobial infections and with mitochondrially encoded NADH dehydrogenase 4L (MT-ND4L) in monomicrobial infections. In polymicrobial infections, we found correlations between the human pseudogene Ferritin (FTH1P2) and two *S. pyogenes* genes *sagF* (Q1JHQ0) and *sagG* (A2RFD6). *S. pyogenes* genes of known significance are given in Table 2 and the human genes in Table 3. For all human and bacterial gene interactions and their known functions from Uniprot, we refer you to the Additional file 1: Tables S1, S2, S3 and S4.

**Table 2:** Overview of the most relevant *S. pyogenes* genes obtained from the analysis of host-pathogen gene-gene association networks. *S. pyogenes* could be identified in both NSTI types, mono- and polymicrobial

Gene	Name	NSTI type	Description	Reference
Q1J9L2	Sic1	Mono	Complement inhibitor protein	[36]
Q1JK93	MGAS9429_Spy1542	Mono	Upstream to gene encoding EndoS (modification of IgG antibodies)	[37]
H8HD54	MGAS10270_Spy1608	Mono	Downstream to gene encoding EndoS (modification of IgG antibodies)	[37]
J7MBD1	M1GAS476_1767	Mono	Fibronectin-binding protein (Fba)	[38]
Q99Z78	MurA2	Mono	Peptidoglycan biosynthesis pathway	[39]
A2RGM6	STAB902_09315	Mono	Mediator of bacterial signal transduction	[40]
F5U6Q2		Mono	Immunogenic secreted protein (Isp)	[41, 42]
P0C0H1	HasA	Mono	Hyaluronic acid capsule (important virulence factor)	[43]
Q7CFC6	SpyM3_0408	Mono/Poly	Part of ScfAB-operon	[44]
Q9ZHG8	Lbp	Poly	Adhesion to epithelial cells	[45]
Q1JHQ0	SagF	Poly	Part of the genes that encode for Streptolysin S-operon	[46]
A2RFD6	SagG	Poly	Part of the genes that encode for Streptolysin S-operon	[46]

**Table 3:** Overview of the most relevant human genes obtained from the analysis of host-pathogen gene-gene association networks

Gene	Name	NSTI type	Description	Reference
ZFN354B	Zinc-finger protein	Mono	Transcription regulation	[42]
LRRFIP1	GC-binding factor 2	Mono	Regulation of TNF expression	[42]
CD55	CD55	Mono	Complement decay-accelerating factor	[42]
MT-ND4L	Mitochondrially encoded NADH	Mono	Catalyses electron transfer from NADH	[42]
COL3A1	Collagens	Mono	Structural proteins in the ECM	[47, 48]
COL5A1	Collagens	Mono	Structural proteins in the ECM	[49]
COL6A2	Collagens	Mono	Structural proteins in the ECM	[50, 51]
FTH1P2	Ferritin	Poly	Intracellular iron storage	[52]
KYNU	Kynureninase	Poly	Biosynthesis of NAD cofactors	
CXCL5	C-X-C motif chemokines	Poly	Important role in inflammation	[53–55]
CXCL9	C-X-C motif chemokines	Poly	Important role in inflammation	[53–55]
TGFBI	Kerato-Epithelin	Poly	Cell adhesion & ECM organisation	[42]
SLC11A1	Solute carrier family 11	Poly	Iron metabolism & host resistance	[42]
TAF1D	TBP-associated factor 1D	Poly	Component of transcription factor complex	[42]
TMSB4X	Thymosin Beta - 4	Poly	Organisation of cytoskeleton and actin monomers	[42]

Different genes were associated to different NSTI types, mono- and polymicrobial

### Host-pathogen gene association networks at the patient level

**Fig. 4:**
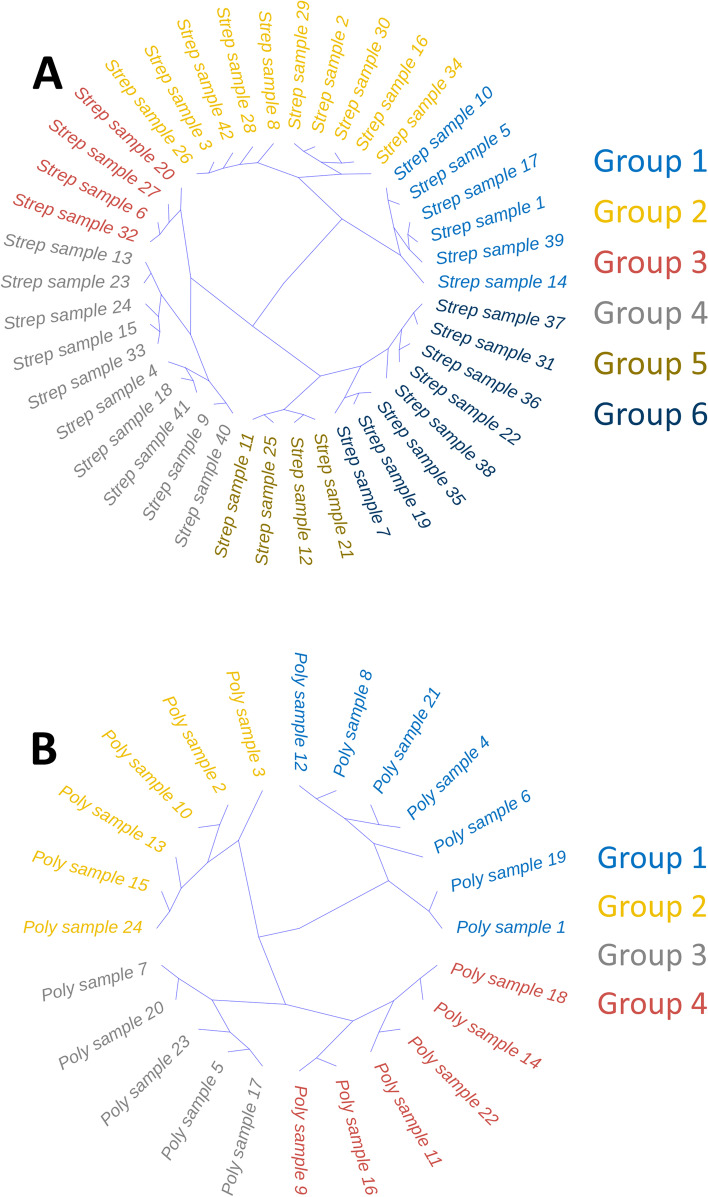
Hierarchical clustering of single sample networks, *i.e*. network derived at the patient level as perturbation networks (see Additional File 1 Section S1.10). A) Clustering of single sample networks from Monomicrobial (*S. pyogenes*) NSTI samples (see Discussion in Section 4.4 B) Clustering of single sample networks from Polymicrobial NSTI samples (see Discussion in Section 4.5)

We used the patient-specific gene-gene correlations to characterise host-pathogen response at a patient level and to stratify patients based on such responses. Patient clusters based on single-sample network edges are shown in Fig. 4 for streptococcal (monomicrobial) (A) and polymicrobial (B) NSTI.

In the case of *S. pyogenes* NSTI, we found 6 distinct groups with 4 to 10 patients each, while for the polymicrobial NSTI, we individuated 4 distinct groups with 5 to 7 patients. For each one of these groups, we retrieved the top 10 most relevant host-pathogen gene associations, characterising the particular response to the infection of each patient group. These are shown in Table 4 and Table 5 for *S. pyogenes* (monomicrobial) and polymicrobial NSTI, respectively. For the polymicrobial case, the top relevant associations involve genes that were mapped to five bacteria species namely *S. pyogenes*, *E. coli*, *P. asaccharolytica*, *P. micra* and *P. oris*. We were unable to ascertain associations between the groups and clinical outcomes of patients with significant statistical power due to the lack of sufficient samples per group.

**Table 4:**
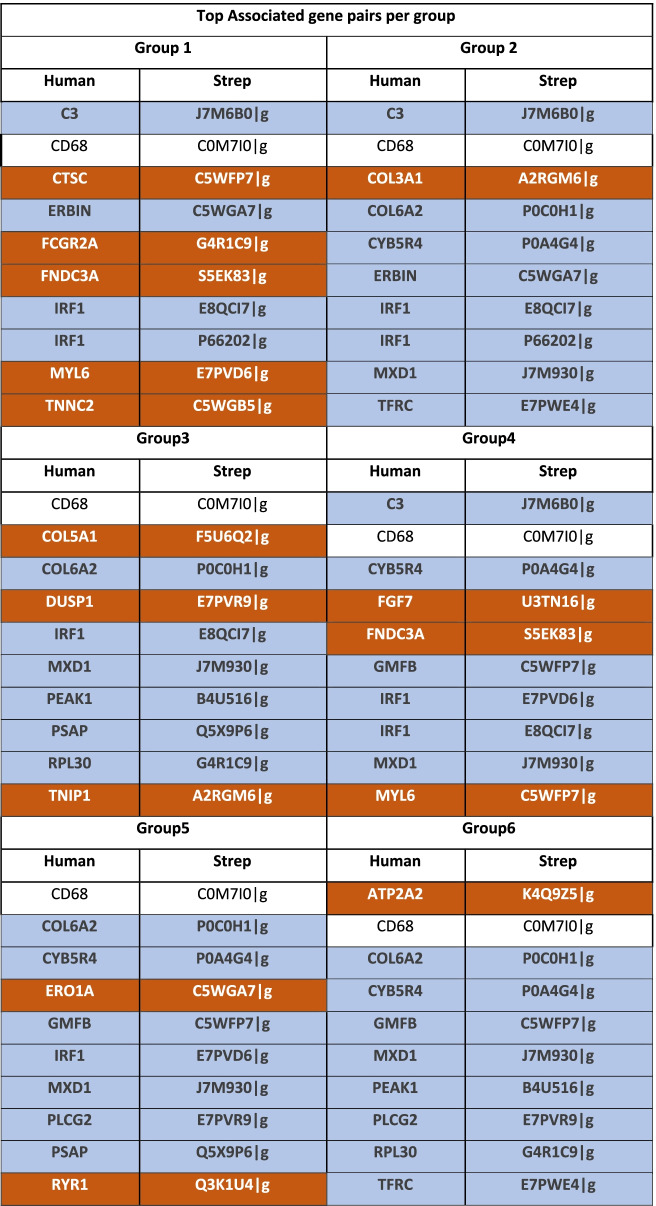
List of the top associated gene pairs for the four patient groups obtained by hierarchical clustering of single sample networks in the case of monomicrobial (*S. pyogenes*) NSTI

Gene associations highlighted in orange are unique for each group and those highlighted in blue occur in 2 or more groups. Unhighlighted gene associations are present in all groups

**Table 5:**
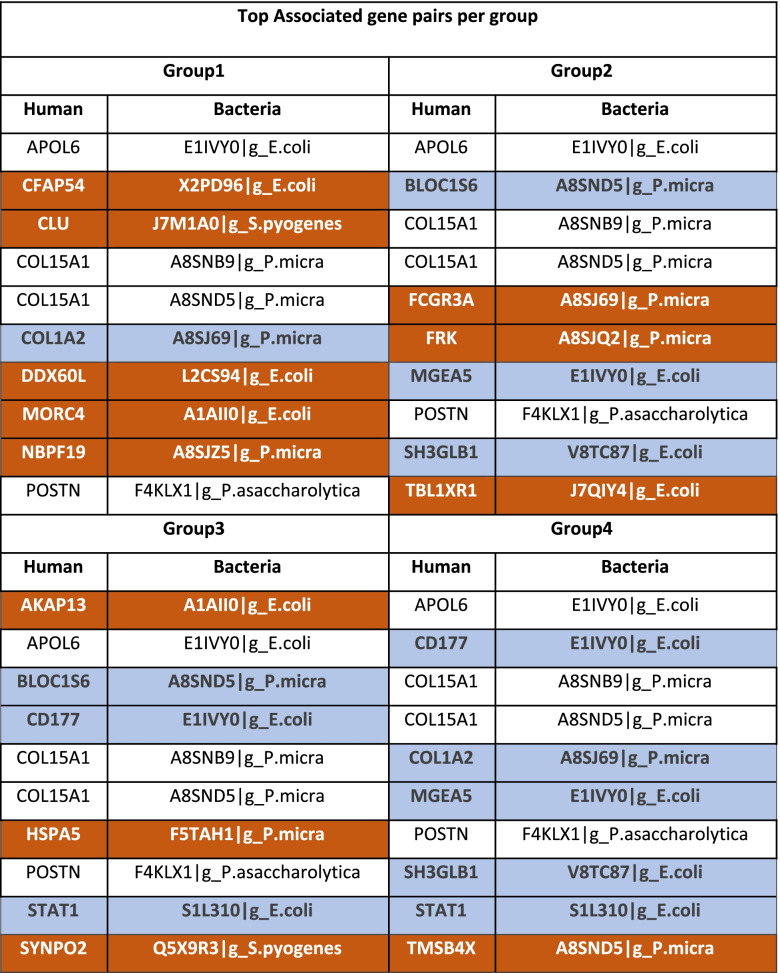
List of the top associated gene pairs for the four patient groups obtained by hierarchical clustering of single sample networks in the case of polymicrobial NSTI

Gene associations highlighted in orange are unique for each group and those highlighted in blue occur in 2 or more groups. Unhighlighted gene associations are present in all groups

## Discussion

**Fig. 5:**
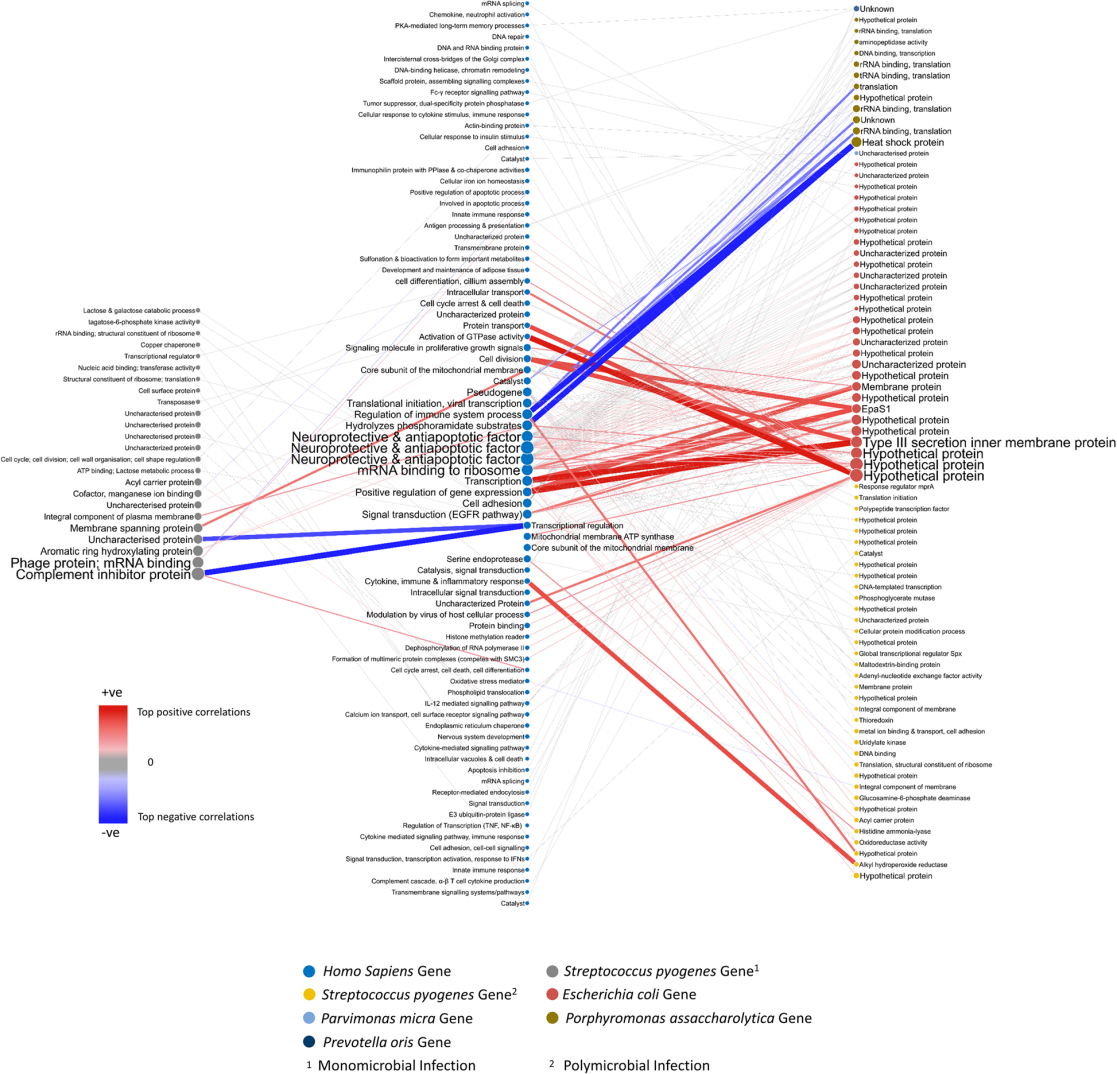
Figure of protein functions associated to the host-pathogen gene expression profiles derived from Dual RNA-seq of tissue biopsies of NSTI patients represented by the interactome network given in Fig. 2. The central column contains human genes, the left column contains *S. pyogenes* genes (i.e. bacterial genes found to be associated with human genes in monomicrobial NSTI), the right column contains genes from several bacterial species (i.e. bacterial genes found to be associated with human genes in polymicrobial NSTI). Nodes are colour coded by bacterial species. The node size is proportional to the node degree (connectivity, i.e. the number of associated genes; see Additional File 1, Equation S3). Red edges indicate positive partial correlation; blue edges indicate negative partial correlation; the colour intensity and the edge width are proportional to the value of the partial correlations

We explored the differences in the host-pathogen transcriptional responses at both the population and individual levels. At the global level, we investigated the differences between the interactomes associated with *S. pyogenes* and polymicrobial NSTI, across the entire cohort of NSTI patient tissue samples. The functions associated with the corresponding proteins in the interactome are shown in Fig. 5. To model the phenotypic heterogeneity observed in the host-pathogen interactions and dynamics at the patient level, we constructed patient-specific interactome networks. Our results provide further insights into the molecular mechanisms underlying the pathophysiology at the tissue site of infection in mono- and polymicrobial NSTIs albeit with some limitations.

### Host-pathogen interactome for Streptococcal NSTI

In the *S. pyogenes* monomicrobial interaction network, the most enriched GO categories for the human genes in the network were cytokine production (GO:0080134) and regulation of response to stress (GO:0001816) suggesting immune system defensive mechanisms in the host response to changes in expression of specific streptococcal genes. The host heat shock protein (HSPA5) is associated with streptococcal acyl carrier protein (P63443, acpP) which is involved in fatty acid biosynthesis in lipid metabolism. Eraso et al. have demonstrated the selection of mutations in the *fabT* gene, another gene involved in fatty acid biosynthesis during necrotising myositis infections in a non-human primate model [56]

Q1J9L2 is 90% identical to Sic1 [36]. Sic has several different mechanisms of actions, interference with complement and other host defences, and has been proposed to play a significant role in streptococcal infections [57]. It was recently shown that the Sic protein from M1 *S. pyogenes*, a type over-represented among severe invasive cases of NSTI [20], interacts with TLR2 resulting in release of pro-inflammatory cytokines [58]. A study by Kachroo et al. [59] revealed a positive correlation between *sic* and genes involved in the host immune response and inflammation when they examined the dual RNA-seq transcriptomes of *S. pyogenes* and host skeletal muscle from infected non-human primates. In addition, vaccination-induced anti-sic antibodies were effective in bacterial clearance in rabbit, mice, and in an ex vivo whole body assay [60].

Sic has been shown to bind to extracellular histones, a group of danger signals released during necrotising tissue damage. The aggregates formed from this interaction have been shown both in vitro and in co-localised biopsies from NSTIs resulting in the neutralisation of host antimicrobial activity [57]. The study by Frick et al. showed that Sic enhances bacterial survival in an animal model of subcutaneous infection [61]. .The increase in the expression of Sic (Q1J9L2) and its role in the inhibition of complement, accompanied by a downregulation of ZFN354B may be a Streptococcal strategy to evade the host innate immune response.

Different bacteria are known to target steps of host gene expression during pathogenic invasion, potentially as a mechanism to modify the expression of inflammatory genes [62]. The ZFN354B gene is also negatively correlated with the gene MGAS9429_Spy1542 (Q1JK93). Although, the sequence of this gene is unannotated, it is located immediately upstream to the gene encoding EndoS. The gene immediately downstream from the gene encoding EndoS, MGAS10270_Spy1608 (H8HD54), is also found in our analysis correlated with the human gene Neurensin (NRSN1). Even though the exact function of these two gene sequences is unclear, it should be noted that the protein EndoS displays endoglycosidase activity on immunoglobulin G (IgG) by hydrolysing the chitobiose core of the asparagine-linked glycan. EndoS modification of IgG antibodies results in impaired Fc-dependent effector function involved in phagocytic killing and elimination of antibody-antigen complexes from circulation [37, 63]. The strong connectivity of Sic, the EndoS-region, and others in the monomicrobial NSTI networks underlines the importance of the immune evasive strategies in *S. pyogenes* infections. Interestingly, we find that the transcription of both these virulence factors is abated by clindamycin, lending support to contemporary guidelines advocating adjunctive clindamycin treatment in streptococcal NSTIs [64].

LRRFIP1 gene was found associated with the *S. pyogenes* gene M1GAS476_1767 (J7MBD1) encoding Fba. Fba is a cell-wall-anchoring, surface-located protein that is found in M-type 1, 2, 4, 22, 28 and 49. These M-types constitute 55 out of the 95 sequenced isolates in the INFECT study [18]. On studying the effects of Fba in relation to the bacterial invasion of and adhesion to HEp-2 cells, Terao et al. inferred that the presence of both Fba and M-protein are required for the most efficient bacterial adhesion and invasion. In addition, the report showed that a Fba mutant displayed lower mortality in a murine skin infection model [38].

### Host-pathogen interactome for polymicrobial NSTI

In the *S. pyogenes* subnetwork from the polymicrobial interaction network, the most enriched GO category for the human genes was GO:0034097 (Response to Cytokine) with 6 genes (STAT1, IL18R1, KYNU, POSTN, CXCL9, CXCL5) out of 20 annotated with this GO term. IL18R1, the IL18 receptor complex and the chemokine CXCL5 are associated with bacterial Spx which has an important role in growth, general stress protection and biofilm formation in *S. aureus* [65]. Therefore, the transcriptional response to cytokines in the host is associated with a change in the regulation of transcription in *S. pyogenes* that may impact its ability to form biofilm and modulate its response to stress.

The human gene Ferritin heavy chain 1 Pseudogene 2 (FTH1P2) was found associated with three *S. pyogenes* genes *lbp* (Q9ZHG8), *sagF* (Q1JHQ0) and *sagG* (A2RFD6). Although *fth1p2* is a pseudogene, studies have recognised it to compete with *fth1*, the main intracellular iron-storage protein in the cytoplasm [52]. The study by Terao et al. on adhesion of *S. pyogenes* to the HEp-2 cells showed that the absence of Lbp significantly lowered the efficiency of adhesion to epithelial cells [45]. More studies have indicated that the primary function of Lbp is as a zinc-scavenger and referred to the gene as *adcA* [66, 67]. The genes *sagF* and *sagG* are two out of the nine genes that form the Streptolysin S-operon. The toxin Streptolysin S has been shown to be responsible for the Beta-haemolysis observed by *S. pyogenes* and has also been implicated in NSTI pathogenesis [46, 68–70]. The association of these *S. pyogenes* genes to *fth1p2* is unclear.

Enrichment of immune-related host genes was also found in 6 out of the 12 host genes in the *P. asaccharolytica* subnetwork which are annotated with the GO category - Immune system process (GO:0002376). *E. coli* gene interactions with host genes did not reveal any enrichment of genes with immune-related functions. In this subnetwork, enrichment of host genes in cellular localisation and intracellular protein transport was observed and several of the host genes have roles in transcriptional regulation. The majority of the co-expressed bacterial genes in the *E. coli* subnetwork are uncharacterised proteins.

In the *P.asaccharolytica* subnetwork from the polymicrobial interaction network, the most highly connected human gene is KYNU which is correlated with eight *P. asaccharolytica* genes, five of which code for ribosomal proteins (*rpmF*, *rplU*, *rpsR*, *rplF*, *rpsG*), one is the small heat shock protein Hsp20 (encoded by Poras_0808) and two are proteins of unknown function [71]. Heat shock proteins are chaperones that can interfere with the uncontrolled protein unfolding that occurs under stress, such as the immune response of the host [72]. An increased kynurenine pathway activity has been linked to inflammation and immune activation and has been implicated in diverse diseases such as depression and cancer [73–75].

Expression of the host gene, TGFBI, an important cytokine with broad regulatory role in the immune system was also positively correlated with five ribosomal genes (*rplU*, *rpsR*, *rplF*, *rpsG*, *rpsO*) in *P. asaccharolytica*. Two other host genes, SIAE and HINT3, are associated with the ribosomal genes (*rplU*, *rplF*) and Hsp20 in this species. SIAE has been functionally associated with autoimmune diseases and preeclampsia [76], and although it has a role in the immune system, it has not previously been studied in the context of an infection. The host gene, SLC11A1, is also associated with the ribosomal gene *rplF*. SLC11A1 controls natural resistance to infection with intracellular parasites. Pathogen resistance involves sequestration of Fe(2+) and Mn(2+), cofactors of both prokaryotic and eukaryotic catalases and superoxide dismutase, not only to protect the macrophage against its own generation of reactive oxygen species, but also to deny the cations to the pathogen for the synthesis of its protective enzymes.

### Differences between host-pathogen interactions in streptococcal infections in monomicrobial and polymicrobial NSTI

The expression of *S. pyogenes* gene SpyM3_0408 (Q7CFC6) was found to be correlated with the expression of human genes in both monomicrobial and polymicrobial infections. In monomicrobial infections, it was positively correlated with the gene mitochondrially encoded NADH dehydrogenase 4L (MT-ND4L). In polymicrobial infections, it was associated with the gene TATA Box-binding protein-associated factor 1D (TAF1D). The gene SpyM3_0408 (Q7CFC6) itself is transcribed as part of the three-gene *scfAB*-operon. It was hypothesised by Breton et al. that the *scfAB*-operon plays an integral role in enhancing adaptation and fitness of *S. pyogenes* during localised skin infection and potentially in the propagation to other deeper tissue in a genome-wide Tn-seq analysis to identify *S. pyogenes* genetic determinants necessary for in vivo fitness using a murine model of skin and soft tissue infection. The scfAB-operon is part of *S. pyogenes* core genome, and the gene encodes for a putative transmembrane protein and was found important at the subepithelial site of infection and for the dissemination of into the bloodstream. Homologues of the scfAB-operon are also found in other pathogenic streptococci and closely related gram-positive pathogens [44].

Although cytokine gene expression was associated with *S. pyogenes* gene expression in both polymicrobial and monomicrobial infections, the only common host gene involved was IL18R1. This occurrence of IL18R1 in both types of infections stands to reason as the Random Forest (RF) models built from cytokine concentrations in the study by Palma Medina et al. found IL18 to be the least important cytokine to differentiate between monomicrobial and polymicrobial NSTI [77].

The genes representing chemokines CXCL5 and CXCL9 were only found in the *S. pyogenes* subnetwork in polymicrobial infections and not in monomicrobial infections. Thänert et al. found CXCL9, CXCL10 and CXCL11 to be overexpressed in streptococcal NSTI [18] and a recent study analysing cytokines and chemokines in plasma samples from NSTI patients by Palma Medina et al. found CXCL10 to be a robust biomarker for differentiating between monomicrobial and polymicrobial NSTI infections [77]. In accordance with these studies, our analysis shows a difference in the involvement of these chemokines between monomicrobial and polymicrobial *S. pyogenes* response. The nature of this network analysis renders any information regarding over-expression or under-expression unascertainable.

### Host-pathogen gene associations at the patient level in streptococcal NSTI

Overall, we observed different associations of genes coding for collagen proteins, consistent with the study by Singh et al. which showed that various human pathogens utilise the proteins found in the extracellular matrix (ECM) such as collagen proteins for the invasion of the host. Invasive pathogens infract the basal lamina and degrade the ECM proteins employing various proteases drafted from the host. Pathogens use these abilities to adhere to and invade the host tissue [47].

Group 2 differs from other groups only for perturbation of the association between COL3A1 and A2RGM6. In group 3, we find the association between collagen type V and the gene (F5U6Q2) encoding for a hypothetical protein with high similarity (91.4%) to an immunogenic secreted protein (Isp). This *isp* gene is located immediately downstream of the *ihk-irr* TCS and the gene is highly conserved among *S. pyogenes* strains [41]. The function of Isp remains elusive but Kachroo et al. showed that the *isp* gene contributed to virulence in a necrotising myositis model in non-human primates. Kachroo et al. also postulated the potential role of *isp* in cell-wall metabolism based on the CHAP domain located at the carboxy terminus [59].

The P0C0H1 (HasA) is required for hyaluronic acid capsule. The capsule represents an important virulence factor of *S. pyogenes* [43]. We find this gene associated with collagen VI in groups 2, 3, 5 and 6. Despite Collagen VI having antimicrobial properties [78], it has been shown to be a target of adherence by *S. pyogenes* for persistent infections and inducing invasions [79]. Immunodetection, in vitro binding assays and electron microscopy have shown *S. pyogenes* to have a strong affinity to Collagen VI and evolved adhesins with the ability to mediate interactions between *S. pyogenes* and the host [79]. Studies have reported the possibility of hyaluronic capsule to be a major virulence determinant and is also listed in the PATRIC database as a known virulence factor [80]. Dinkla et al. [81] demonstrated that in rheumatic fever, *S. pyogenes* had the unique capability to bind and aggregate membrane collagen type IV via the M3 protein or hyaluronic acid capsule in M18 serotype. It has also been shown that the upregulation of hyaluronic acid by *S. pyogenes* in blood is used as a mechanism to mask surface immunogenic determinants and evade antigen-specific antibodies to avoid bacterial death in blood. Dinkla et al. [82] managed to demonstrate that only the *S.* pyogenes abundant in hyaluronic acid capsule were capable of surviving in human blood containing high levels of antibodies directed against highly conserved bacterial surface proteins. This association may point to a mode of entry and immune evasion by *S. pyogenes*.

### Host-pathogen gene associations at the patient level in polymicrobial NSTI

Group 4 differs from other groups only for perturbation of the association between the host gene TMSB4X and the *P. micra* gene A8SND5. The TMSB4X gene plays an important role in the organisation of the cytoskeleton and binds to and sequesters actin monomers (G actin) inhibiting actin polymerisation. A common target of bacterial pathogens is the host cell actin cytoskeleton, a dynamic system of filaments that is central to shape determination, movement, phagocytosis and intracellular trafficking [83]. After invasion, some pathogens remain within a membrane-bound compartment and target actin to subvert membrane trafficking [83] by polymerising actin on their surface and use filament assembly to power intracellular actin-based motility, generating actin comet tails that trail the moving bacteria [83–85]. The associated *P. micra* gene A8SND5 (*rplC*) highlights the importance of the expression of ribosomal proteins to increase bacterial protein synthesis indicating a possible remodulation of this interplay between host and pathogen.

The association of the *E. coli* gene encoding for protein E1IVY0 and several human genes are present in all 4 groups. The E1IVY0 is an uncharacterised protein with high similarity (86%) to a serine acetyl transferase from *Pantoea ananatis*, a plant pathogenic gram-negative, facultatively anaerobic gamma proteobacterium which has been shown to help the bacterium survive oxidative stress conditions [86, 87].

### Limitations of the study

Limitations of this study include the (relatively) small number of samples, heterogeneity among patients with respect to their comorbidities, the time of infection and treatments, along with the differences in biopsy sampling, type of tissue, depth of infection and lack of longitudinal samples. Many of these limitations are unavoidable as the biopsies are obtained at a timing when surgery is clinically indicated and in areas where the tissue pathology warrants surgical removal. However, using important quality aspects such as the collection of biopsies by dedicated teams of clinicians using standardised SOPs, a prospective observational study design similar in all participating clinical centres and the employment of highly stringent statistical approaches strengthen this study.

## Conclusions

Using a systems biology approach to explore host-pathogen interactions in NSTIs, we postulated several data-driven hypotheses which could be further evaluated experimentally. In attempting to elucidate the mechanisms underlying the progression and proliferation of NSTI infections, this study highlights the heterogeneity in the host-pathogen interactomes and strengthens the rationale for a personalised approach in the clinical management of NSTI patients. Furthermore, the identification of pivotal pathogenetic mechanisms is the first step towards identifying novel targets for intervention and expanding our therapeutic armamentarium.

## Supplementary Information

**Additional file 1: Section S1.** Extended Methods. **Figure S1.** (A): PCA of gene expression profiles. (B): Random Forest classification using gene expression profiles. **Table S1.** Corresponding protein functions of all interactions between *S.pyogenes* and human genes in monomicrobial infections. **Table S2.** Corresponding protein functions of all interactions between *S.pyogenes* and human genes in polymicrobial infections. **Table S3.** Corresponding protein functions of all interactions between *P.asaccharolytica* and human genes in polymicrobial infections. **Table S4.** Corresponding protein functions of all interactions between *E.coli* and human genes in polymicrobial infections.

## Data Availability

All the data used in this available at https://zenodo.org/record/5744186#.YlUsVdNBxPZ (DOI: 10.5281/zenodo.5744186).

## Abbreviations

AMPs: Antimicrobial peptides
CTGF: Connective tissue growth factor
ECM: Extracellular matrix
GMM: Gaussian Graphical Model
GO: Gene Ontology
HSP: Heat shock protein
IgG: Immunoglobulin G
isp: Immunogenic secreted protein
LIONESS: Linear Interpolation to Obtain Network Estimates for Single Samples
lm: Laminin
NSTI: Necrotising soft tissue infection
PCLRC: Probabilistic Context Likelihood of Relatedness on Correlation
RF: Random forest
