# Supplementary material for: Analysis of host-pathogen gene association networks reveals patient-specific response to streptococcal and polymicrobial necrotising soft tissue infections
Source: BMC Med. 2022 May 4;20:173. doi: 10.1186/s12916-022-02355-8 (PMC9066942; doi:10.1186/s12916-022-02355-8)
Supplement: Supplementary file 1 — Additional file 1: Section S1. Extended Methods. Figure S1. (A): PCA of gene expression profiles. (B): Random Forest classification using gene expression profiles. Table S1. Corresponding protein functions of all interactions between S.pyogenes and human genes in monomicrobial infections. Table S2. Corresponding protein functions of all interactions between S.pyogenes and human genes in polymicrobial infections. Table S3. Corresponding protein functions of all interactions between P.asaccharolytica and human genes in polymicrobial infections. Table S4. Corresponding protein functions of all interactions between E.coli and human genes in polymicrobial infections. [file 12916_2022_2355_MOESM1_ESM.docx]

**Additional File 1: Analysis of host-pathogen gene association networks reveals patient-specific response to streptococcal and poly-microbial necrotizing soft tissue infections**

Sanjeevan Jahagirdar^1^, Lorna Morris^2^, Nirupama Benis^1^**^†^**, Oddvar Oppegaard^3^, Mattias Svenson^4^, Ole Hyldegaard^5,6^, Steinar Skrede^3,7^, Anna Norrby-Teglund^4^, INFECT Study group^#^, Vitor A. P. Martins dos Santos^1,2^ and Edoardo Saccenti^1*^

^1^Laboratory of Systems and Synthetic Biology, Wageningen University & Research, Stippeneng 4, 6708 WE, Wageningen, the Netherlands

^2^Lifeglimmer GmbH, Markelstraße 38, 12163 Berlin, Germany

^3^Department of Medicine, Division for infectious diseases, Haukeland University Hospital, Bergen Norway

^4^Center for Infectious Medicine, Department of Medicine, Karolinska Institutet, Karolinska University Hospital, Huddinge, Sweden

^5^Department of Anesthesia, Centre of Head and Orthopaedics, Copenhagen University Hospital, Rigshospitalet, Copenhagen, Denmark

^6^Department of Clinical Medicine, University of Copenhagen, Denmark.

^7^Department of Clinical Science, University of Bergen, Bergen, Norway

**^†^**Current affiliation of Nirupama Benis: Department of Medical Informatics, Amsterdam Public Health Research Institute, Amsterdam UMC, University of Amsterdam, Meibergdreef 9, Amsterdam, Netherlands

^#^INFECT study group (Trond Bruun, Eivind Rath, Torbjørn Nedrebø, Per Arnell, Anders Rosen, Morten Hedetoft, Martin B. Madsen, Mattias Svensson, Johanna Snäll, Ylva Karlsson, & Michael Nekludov)

*To whom correspondence should be addressed:

Edoardo Saccenti ([edoardo.saccenti@wur.nl](mailto:edoardo.saccenti@wur.nl)) Laboratory of Systems and Synthetic Biology, Wageningen University & Research, Stippeneng 4, 6708 WE Wageningen, the Netherlands. Tel: +31 (0) 317 486948

**S1. Extended Methods**

**S1.1 Study design**

Tissue biopsies and plasma samples on the day of hospital admission (day 0) were obtained from patients diagnosed with NSTI and admitted to Karolinska University Hospital in Stockholm, Copenhagen University hospital, Blekinge County Council Hospital in Karlskrona, Sahlgrenska University Hospital in Gothenburg and Haukeland University Hospital in Bergen in the framework of the EU project INFECT (https://permedinfect.com/projects/peraid/).

Diagnosis of NSTI was based on the presence of necrotic or deliquescent soft tissue with widespread undermining of the surrounding tissue. Patients were excluded in absence of reports of necrotic or deliquescent tissue. More details on patient characteristics and study design can be obtained from^1^.

**S1.2 Ethical Considerations**

The INFECT study was conducted in accordance with the Declaration of Helsinki and was approved by the regional Ethical Review Board at the Karolinska Institutet in Stockholm, Sweden (Ethics Permits: 2012/2110-31/2), the National Committee on Health Research Ethics in Copenhagen, Denmark (Ethics permits: 1151739), the regional Ethical Review Board in Gothenburg, Sweden (Ethics permits: 930-12) and Bergen, Norway (2012/2227/REC West). All experiments were performed in accordance with the approved ethics applications specified above. All patients provided written informed consent. The INFECT study is registered at ClinicalTrials.gov (NCT01790698).

**S1.3 Experimental Methods**

**S1.3.1 RNA-seq sample preparation and sequencing**

This study makes use of RNAseq samples used in ^1,2^ plus additional data not available at the time. All samples were handled and processed as described in ^1,2^.

**S1.3.2 Sample Selection**

In line with the study by Thänert et al., we retained all those subjects/samples for which dual RNA-seq data (i.e. transcriptomics data for both host and pathogen) was available together with 16S bacterial rRNA gene sequencing data collected on the day of hospital admission: this results in 81 samples available for analysis (Figure 6).

We followed the classification established by Thanert et al.^2^ who assigned the 81 samples to 5 different types of infection based on their associated bacterial composition according to 16S rRNA gene sequencing, namely *Staphylococcus* (n = 5), *Streptococcus* (n = 42), *Polymicrobial* (n = 25), *Escherichia/Shigella/Bacteroides* (n = 4) and other (n = 5). Classification was based on average-linkage hierarchical agglomerative clustering using the relative abundance of the identified bacterial communities. The optimal number of clusters in the resulting sample dendrogram was determined using the J-index and distinct specimen clusters were defined to represent different types of NSTIs. This information was obtained from the Supplementary Data 3 from^2^.

The sample size for *Staphylococcus, Escherichia*/*Shigella*/*Bacteroides* and other was not large enough to build robust correlation networks, thus we focused on the two larger groups, namely samples from patients with Streptococcal and polymicrobial NSTI. We indicate with Hstrep and Hpoly the matrix of human gene expression measured on biopsies obtained from patients with diagnosis streptococcal NSTI and polymicrobial NSTI, respectively; we indicate with Bstrep and Bpoly the matrix of bacterial gene expression for streptococcal NSTI and polymicrobial NSTI, respectively.

**S1.4 Bioinformatics analysis**

RNA sequences were mapped against both human and bacterial genomes to obtain gene expression of both the host and resident pathogens. Quality control was performed with the tool FASTQC^3^. The mapping tool Kallisto^4^ was used to map the sequences against the human genome (GRCh38 release 91). The resulting read counts, from TSV files, were loaded into R using the package tximport^5^.

The same sequences were also mapped against several bacterial genomes using the published pipeline HUMAnN2 (version 0.11.1)^6^ which uses a series of tools to map RNA sequences to bacterial proteins from the database UniRef^7^. HUMAnN2 uses a tiered search for taxonomic profiling. Firstly, it searches for a pre-selected set of marker genes (from the MetaPhIAn2 database) unique for each species. Secondly, it maps all the remaining reads to the pangenomes of the detected species. Thirdly, the remaining unclassified reads are mapped to a protein sequence database (DIAMOND). We used the default coverage threshold in HUMAnN2 in order for a sequence in a particular species to be detected. Alignments are not considered if they do not pass the default coverage threshold. We opted to use the recommended database UniRef90. Uniref90 identifiers have the form UniRef90_{ID}, where ID is a UniProtKB accession or UniParc identifier of the representative sequence from the cluster of 90% identical sequences referred to by UniRef90_{ID}. For ease of discussion and visualization in this manuscript, we refer to the ID part only and use functional annotation from Uniprot where available to describe these sequences.

The mapped human and bacterial data were post-processed and filtered before integration with each other. Filters were set for the level of gene expression and this threshold had to be met in at least 10 samples to focus on gene expression patterns in host and pathogen that are predominant in several patients. Since not all genes are expressed or can be in all samples, we needed to ensure that a gene was expressed in a sufficiently large number of samples to avoid spurious associations. We found that 10 samples were sufficient to ensure robustness of the results. In addition, genes mapped from *E. coli* were also removed from samples classified as ‘*Streptococcus*’ according to 16S sequencing. The final data sets for the *Streptococcus* monomicrobial classified samples contained 680 human genes and 721 bacterial Uniref90 sequences, and the poly-microbial classified samples contained 680 human genes and 703 bacterial Uniref90 sequences. The gene-level abundance was calculated as reads per kilobase units (RPKU), as defined by HUMAnN2.

**S1.5 Data Transformation**

Data was transformed to stabilise the variance of the data. Gene expression values were transformed taking the square root of the original values.

**S1.6 Predictive modelling**

The Random Forest (RF) algorithm was used to classify monomicrobial and polymicrobial patients on the basis of human gene expression profiles^11^. To reduce the potential bias due to an unbalanced number of subjects/samples per group, we imposed a number of k=100 resampling, considering the 85% of data to retain for each compared group. Accuracy, sensitivity, specificity, and related 95% CI of all performed models were assessed according to the standard definitions and were determined by means of permutated test (k=1000 times). For all calculations, the “randomForest” function, implemented in the R package Random Forest, was used to grow a decision forest composed of 1000 trees. Default parameters were used.

**S1.7 Gene-Gene Association Network Inference**

Association networks between human and bacterial genes were built using the Probabilistic Context Likelihood of Relatedness on Correlation (PCLRC) algorithm^12^ which is based on the original CLR algorithm^13^ and has been shown to be robust against variation in sample size and noise^14^. In the current study, we replaced standard correlations between two molecular features *i* and *j* (either metabolites or genes) with partial correlations obtained using a Gaussian Graphical Model (GMM). The PCLRC algorithm uses resampling to estimate robust correlation based on the Context Likelihood of Relatedness approach which estimates the relevance of the associations between two features by considering background associations. The PCLRC returns a probability matrix P, containing the likelihood 0 < *p_ij_* < 1 of each observed association *r_ij_* between each gene pair.

All p-values were corrected for multiple testing using the Benjamini & Hochberg method^15^. Only corrected P-values (P_adjust) smaller than 0.05 were retained in the analysis to give us a partial correlation network of genes filtered for only the most significant associations. Significant associations *r_ij_* between the *i*-th host and *j*-th pathogen gene were defined in equation 1. Default parameters were used (number of resampling iterations Niter = 1000; the fraction of the samples to be considered at each iteration fraction = 0.75 and fraction of the total predicted interactions to be kept at each iteration rank.thr = 0.3). All networks are undirected and represented as an *m* × *m* adjacency matrix M, populated by association (edges) between genes *i* and *j* (nodes). For each node in the *m*×*m* network(s) we calculated the node degree (connectivity) as shown in Equation 2.

**S1.8 Estimation of partial correlations using Gaussian Graphical Model**

Partial correlations were estimated using a Gaussian Graphical Model (GGM) as implemented in the GeneNet R package^16,17^. GeneNet allows estimating a GGM from a small sample of high-dimensional data in a computationally and statistically efficient way. It uses an analytic shrinkage estimation of covariance and partial correlation matrices and performs optimal model selection based on local false discovery rate multiple testing. The edges (i.e., the associations) to be included in the final association network are selected using a computational algorithm depending on the relative values of the pairwise partial correlations. For more details on GeneNet implementation, we refer to the original publication^17^.

We used partial correlation since partial correlations represent direct associations, while standard correlation analyses do not distinguish between indirect and direct associations, thus partial correlations are more likely to represent primary dependencies and causative links between host and pathogen transcripts. Partial correlations are in general much smaller in value than standard correlations (see for instance^18^).

The association between two nodes *i* and *j* was defined as

$r_{ij}=\left\{ \begin{aligned} r_{ij} ifP_{adjust}\leq0.05 \\ \boldsymbol{0 otherwise} \end{aligned} \right.$ **(S1)**

where, $r_{ij}$ is the association between genes i and j and $P_{adjust}$ is the corrected P-value for multiple tests.

The connectivity (node degree) of a gene is defined as

${conn}_{j}=\sum_{i=1}^{p} r_{ij}$ **(S2)**

**S1.9 Functional Analysis**

TopGO R-package v2.42.0 was used for functional category enrichment analysis^8^ using the human genes from each bacterial sub-network as the target sets of interesting genes and the list of all genes from human genome build GRCh38.p12 downloaded from Ensembl Biomart^9^ as the background set. The Biological Process ontology from Gene Ontology was used^10^ and the Fisher’s exact test was selected to calculate statistical significance of enrichment for the genes of interest.

**S1.10 Inference of host-pathogen gene association networks at the patient level**

We used the Linear Interpolation to Obtain Network Estimates for Single Samples (LIONESS) method to infer the single sample networks^19,20^. For sample q (containing gene expression profiles for host and pathogen) out of n samples the corresponding LIONESS single sample network is obtained as

$E^{q}=n\left( E^{\alpha}-E^{\left( \alpha-q \right)} \right)+E^{\left( \alpha-q \right)}$(S3)

where, $E^{q}$ is the single sample network of the $q^{th}$ sample, $E^{\alpha}$ is the aggregate network constructed from all the samples and $E^{(\alpha-q)}$ is the network constructed from all samples excluding the $q^{th}$ sample.

The networks have been estimated using the same approach described in Network Inference section. Estimation was done separately for streptococcal and polymicrobial NSTI. Note that the aggregate network corresponds to the host-pathogen gene-gene association networks described in the Network Inference section.

**S1.11 Clustering of patients based on single-sample networks**

Each m × m single-sample network can be reduced to a ½m(m-1) × 1 vector containing the perturbations (edges) of the host-pathogen gene correlation. We collapsed these vectors in two matrices of size 2556 × 42 and 2691 × 25. For each matrix pairwise distances (Euclidean) among samples were calculated, and hierarchical clustering was applied using the Ward linkage method^21^.

**1.12 Selection of most relevant single sample network edges**

**Selection of most relevant single sample network edges**

We defined the relevance $A_{ij}$ of each single-sample network edge (describing the perturbation of the association between host gene *i* and pathogen gene *j*) for each group G of patients defined by applying clustering on the single-sample edges as

$A_{ij}=\sum_{s=1}^{n} e_{ij}^{s}$ (S4)

where *e_ij_* is the *i,j*-th edge in the single sample network for the *q*-th subject in group G; the sum runs on the 1,2,...,*n*(G) subjects in group G. Single-sample network edges were then ranked based on per each group. For each group we retained the 10 most relevant edges.

**References**

1. Madsen, M. B. *et al.* Necrotizing soft tissue infections - a multicentre, prospective observational study (INFECT): protocol and statistical analysis plan. *Acta Anaesthesiol. Scand.* **62**, 272–279 (2018).

2. Thänert, R. *et al.* Molecular profiling of tissue biopsies reveals unique signatures associated with streptococcal necrotizing soft tissue infections. *Nat. Commun.* **10**, 3846 (2019).

3. Andrews Simon, Krueger Felix, Segonds-Pichon Anne, Biggins Laura, Krueger Christel, W. S. FastQC. (2010).

4. Bray, N. L., Pimentel, H., Melsted, P. & Pachter, L. Near-optimal probabilistic RNA-seq quantification. *Nat. Biotechnol.* **34**, 525–527 (2016).

5. Soneson, C., Love, M. I. & Robinson, M. D. Differential analyses for RNA-seq: transcript-level estimates improve gene-level inferences. *F1000Research* **4**, 1521 (2016).

6. Franzosa, E. A. *et al.* Species-level functional profiling of metagenomes and metatranscriptomes. *Nat. Methods* **15**, 962–968 (2018).

7. Suzek, B. E., Huang, H., McGarvey, P., Mazumder, R. & Wu, C. H. UniRef: comprehensive and non-redundant UniProt reference clusters. *Bioinformatics* **23**, 1282–1288 (2007).

8. Alexa, A. & Rahnenfuhrer, J. topGO: Enrichment Analysis for Gene Ontology. R package version 2.42. 0 (2020). (2020).

9. Kinsella, R. J. *et al.* Ensembl BioMarts: a hub for data retrieval across taxonomic space. *Database* **2011**, bar030–bar030 (2011).

10. Consortium, G. O. The gene ontology resource: 20 years and still GOing strong. *Nucleic Acids Res* **47**, D330--D338 (2019).

11. Breiman, L. Random forests. *Mach. Learn.* **45**, 5–32 (2001).

12. Saccenti, E., Suarez-Diez, M., Luchinat, C., Santucci, C. & Tenori, L. Probabilistic networks of blood metabolites in healthy subjects as indicators of latent cardiovascular risk. *J. Proteome Res.* **14**, 1101–1111 (2015).

13. Faith, J. J. *et al.* Large-Scale Mapping and Validation of Escherichia coli Transcriptional Regulation from a Compendium of Expression Profiles. *PLoS Biol.* **5**, e8 (2007).

14. Jahagirdar, S., Suarez-Diez, M. & Saccenti, E. Simulation and Reconstruction of Metabolite–Metabolite Association Networks Using a Metabolic Dynamic Model and Correlation Based Algorithms. *J. Proteome Res.* **18**, 1099–1113 (2019).

15. Benjamini, Y. & Hochberg, Y. Controlling the False Discovery Rate: A Practical and Powerful Approach to Multiple Testing. *J. R. Stat. Soc. Ser. B* **57**, 289–300 (1995).

16. Opgen-Rhein, R. & Strimmer, K. From correlation to causation networks: a simple approximate learning algorithm and its application to high-dimensional plant gene expression data. *BMC Syst. Biol.* **1**, 37 (2007).

17. Schäfer, J., Opgen-Rhein, R. & Strimmer, K. Reverse engineering genetic networks using the GeneNet package. *Newsl. R Proj. Vol. 6/5, December 2006* **6**, 50 (2006).

18. Altenbuchinger, M. *et al.* A multi-source data integration approach reveals novel associations between metabolites and renal outcomes in the German Chronic Kidney Disease study. *Sci. Rep.* **9**, 13954 (2019).

19. Kuijjer, M. L., Tung, M. G., Yuan, G., Quackenbush, J. & Glass, K. Estimating Sample-Specific Regulatory Networks. *iScience* **14**, 226–240 (2019).

20. Jahagirdar, S. & Saccenti, E. Evaluation of Single Sample Network Inference Methods for Metabolomics-Based Systems Medicine. *J. Proteome Res.* **20**, 932–949 (2021).

21. Rokach, L. & Maimon, O. Clustering Methods. in *Data Mining and Knowledge Discovery Handbook* 321–352 (Springer-Verlag). doi:10.1007/0-387-25465-X_15.

**Figures**


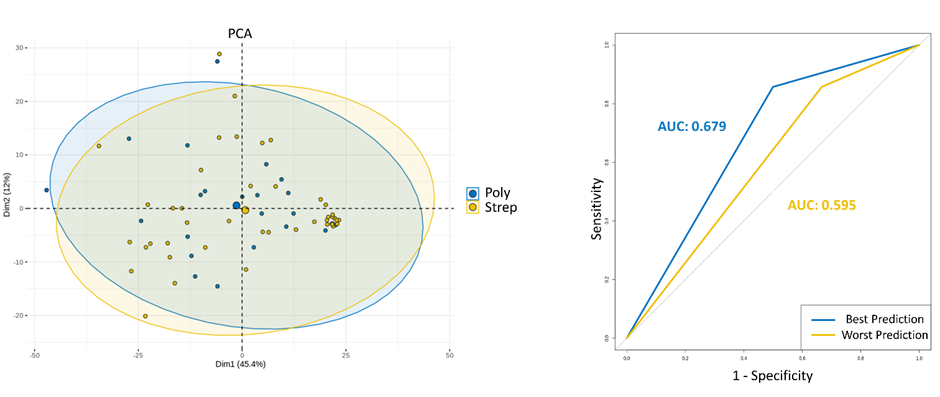


**Figure S1 A)** Principal component analysis of the gene expression profiles of streptococcal (n = 42) and polymicrobial (n=24) NSTI patients. **B)** Random forest classification of NSTI patient using gene expressions profiles. Models are built with cross-validation, and the worst and best model over 100 repetitions are given.

**Tables**

**Table S1: Interactions between human and *S.pyogenes* genes in monomicrobial infections and their corresponding protein functions.**

| **Humangene** | **Pathogengene** | **Human full gene name** | **Human gene Function - from Uniprot** | **Function - from GO terms** | **Pathogen gene short name** | **Pathogen gene full name** | **Pathogen Function** |
| --- | --- | --- | --- | --- | --- | --- | --- |
| STAB1 | Q1J5B9 | Stabilin-1 | Acts as a scavenger receptor for acetylated low density lipoprotein. Binds to both Gram-positive and Gram-negative bacteria and may play a role in defense against bacterial infection. When inhibited in endothelial tube formation assays, there is a marked decrease in cell-cell interactions, suggesting a role in angiogenesis. Involved in the delivery of newly synthesized CHID1/SI-CLP from the biosynthetic compartment to the endosomal/lysosomal system. | cell adhesion, cell-cell signaling, defense response to bacterium, inflammatory response, negative regulation of angiogenesis, receptor-mediated endocytosis | copZ | Copper chaperone copZ | metal ion binding, metal ion transport |
| HSPA5 | P63443 | Endoplasmic reticulum chaperone BiP | Endoplasmic reticulum chaperone that plays a key role in protein folding and quality control in the endoplasmic reticulum lumen (PubMed:2294010, PubMed:23769672, PubMed:23990668, PubMed:28332555). Involved in the correct folding of proteins and degradation of misfolded proteins via its interaction with DNAJC10/ERdj5, probably to facilitate the release of DNAJC10/ERdj5 from its substrate (By similarity). Acts as a key repressor of the ERN1/IRE1-mediated unfolded protein response (UPR) (PubMed:1550958, PubMed:19538957). In the unstressed endoplasmic reticulum, recruited by DNAJB9/ERdj4 to the luminal region of ERN1/IRE1, leading to disrupt the dimerization of ERN1/IRE1, thereby inactivating ERN1/IRE1 (By similarity). Accumulation of misfolded protein in the endoplasmic reticulum causes release of HSPA5/BiP from ERN1/IRE1, allowing homodimerization and subsequent activation of ERN1/IRE1 (By similarity). Plays an auxiliary role in post-translational transport of small presecretory proteins across endoplasmic reticulum (ER). May function as an allosteric modulator for SEC61 channel-forming translocon complex, likely cooperating with SEC62 to enable the productive insertion of these precursors into SEC61 channel. Appears to specifically regulate translocation of precursors having inhibitory residues in their mature region that weaken channel gating. May also play a role in apoptosis and cell proliferation (PubMed:26045166). | ATF6-mediated unfolded protein response, cellular response to antibiotic, cellular response to calcium ion, cellular response to cAMP, cellular response to drug, cellular response to gamma radiation, cellular response to glucose starvation, cellular response to interleukin-4, cellular response to manganese ion, cellular response to nerve growth factor stimulus, cellular response to unfolded protein, cerebellar Purkinje cell layer development, cerebellum structural organization, chaperone cofactor-dependent protein refolding, endoplasmic reticulum unfolded protein response, ER overload response, IRE1-mediated unfolded protein response, luteolysis, maintenance of protein localization in endoplasmic reticulum, negative regulation of apoptotic process, negative regulation of IRE1-mediated unfolded protein response, negative regulation of protein-containing complex assembly, negative regulation of transforming growth factor beta receptor signaling pathway, neuron apoptotic process, neuron differentiation, PERK-mediated unfolded protein response, positive regulation of cell migration, positive regulation of neuron projection development, positive regulation of protein ubiquitination, positive regulation of transcription from RNA polymerase II promoter in response to endoplasmic reticulum stress, posttranslational protein targeting to membrane, translocation, protein folding in endoplasmic reticulum, protein refolding, regulation of ATF6-mediated unfolded protein response, regulation of IRE1-mediated unfolded protein response, regulation of PERK-mediated unfolded protein response, regulation of protein folding in endoplasmic reticulum, response to cocaine, response to methamphetamine hydrochloride, response to unfolded protein, stress response to metal ion, substantia nigra development, toxin transport, ubiquitin-dependent ERAD pathway | acpP | Acyl carrier protein | cytoplasm, acyl carrier activity |
| TFRC | Q1J692 | Transferrin receptor protein 1 | Cellular uptake of iron occurs via receptor-mediated endocytosis of ligand-occupied transferrin receptor into specialized endosomes (PubMed:26214738). Endosomal acidification leads to iron release. The apotransferrin-receptor complex is then recycled to the cell surface with a return to neutral pH and the concomitant loss of affinity of apotransferrin for its receptor. Transferrin receptor is necessary for development of erythrocytes and the nervous system (By similarity). A second ligand, the heditary hemochromatosis protein HFE, competes for binding with transferrin for an overlapping C-terminal binding site. Positively regulates T and B cell proliferation through iron uptake (PubMed:26642240). Acts as a lipid sensor that regulates mitochondrial fusion by regulating activation of the JNK pathway (PubMed:26214738). When dietary levels of stearate (C18:0) are low, promotes activation of the JNK pathway, resulting in HUWE1-mediated ubiquitination and subsequent degradation of the mitofusin MFN2 and inhibition of mitochondrial fusion (PubMed:26214738). When dietary levels of stearate (C18:0) are high, TFRC stearoylation inhibits activation of the JNK pathway and thus degradation of the mitofusin MFN2 (PubMed:26214738). | cellular iron ion homeostasis, cellular response to drug, cellular response to leukemia inhibitory factor, intracellular signal transduction, iron ion transport, membrane organization, negative regulation of apoptotic process, negative regulation of mitochondrial fusion, osteoclast differentiation, positive regulation of B cell proliferation, positive regulation of bone resorption, positive regulation of gene expression, positive regulation of I-kappaB kinase/NF-kappaB signaling, positive regulation of isotype switching, positive regulation of NF-kappaB transcription factor activity, positive regulation of peptidyl-serine phosphorylation, positive regulation of protein localization to nucleus, positive regulation of protein phosphorylation, positive regulation of protein-containing complex assembly, positive regulation of T cell proliferation, receptor internalization, transferrin transport, transport across blood-brain barrier | MGAS10750_Spy1141 | Transcriptional regulator, GntR family | DNA-binding transcription factor activity |
| MAP3K20 | Q1J9L2 | Mitogen-activated protein kinase kinase kinase 20 | Stress-activated component of a protein kinase signal transduction cascade. Regulates the JNK and p38 pathways. Part of a signaling cascade that begins with the activation of the adrenergic receptor ADRA1B and leads to the activation of MAPK14. Pro-apoptotic. Role in regulation of S and G2 cell cycle checkpoint by direct phosphorylation of CHEK2 (PubMed:10924358, PubMed:11836244, PubMed:15342622, PubMed:21224381). Involved in limb development (PubMed:26755636). | activation of JUN kinase activity, activation of MAPKK activity, cell cycle arrest, cell death, cell differentiation, cellular response to gamma radiation, cytoskeleton organization, DNA damage checkpoint, embryonic digit morphogenesis, limb development, mitotic cell cycle checkpoint, positive regulation of apoptotic process, protein phosphorylation, stress-activated MAPK cascade |  |  |  |
| SIAE | A2RFA2 | Sialate O-acetylesterase | Catalyzes the removal of O-acetyl ester groups from position 9 of the parent sialic acid, N-acetylneuraminic acid. | carbohydrate metabolic process, regulation of immune system process |  |  |  |
| HINT3 | A2RFA2 | Histidine triad nucleotide-binding protein 3 | Hydrolyzes phosphoramidate and acyl-adenylate substrates. |  |  |  |  |
| HINT3 | P60965 | Histidine triad nucleotide-binding protein 3 | Hydrolyzes phosphoramidate and acyl-adenylate substrates. |  | lgt | Phosphatidylglycerol--prolipoprotein diacylglyceryl transferase | integral component of plasma membrane, phosphatidylglycerol-prolipoprotein diacylglyceryl transferase activity, lipoprotein biosynthetic process |
| IL1R2 | G4R112 | Interleukin-1 receptor type 2 | Non-signaling receptor for IL1A, IL1B and IL1RN. Reduces IL1B activities. Serves as a decoy receptor by competetive binding to IL1B and preventing its binding to IL1R1. Also modulates cellular response through non-signaling association with IL1RAP after binding to IL1B. IL1R2 (membrane and secreted forms) preferentially binds IL1B and poorly IL1A and IL1RN. The secreted IL1R2 recruits secreted IL1RAP with high affinity; this complex formation may be the dominant mechanism for neutralization of IL1B by secreted/soluble receptors. | cytokine-mediated signaling pathway, immune response, interleukin-1-mediated signaling pathway, negative regulation of cytokine production involved in inflammatory response, negative regulation of interleukin-1 alpha production, negative regulation of interleukin-1-mediated signaling pathway, negative regulation of protein processing |  |  |  |
| IL18R1 | Q99Z78 | Interleukin-18 receptor 1 | Within the IL18 receptor complex, responsible for the binding of the proinflammatory cytokine IL18, but not IL1A nor IL1B (PubMed:8626725, PubMed:14528293, PubMed:25261253, PubMed:25500532). Involved in IL18-mediated IFNG synthesis from T-helper 1 (Th1) cells (PubMed:10653850). Contributes to IL18-induced cytokine production, either independently of SLC12A3, or as a complex with SLC12A3 (By similarity). | cellular response to cytokine stimulus, immune response, inflammatory response, interleukin-18-mediated signaling pathway, natural killer cell activation, negative regulation of cold-induced thermogenesis, positive regulation of interferon-gamma production, positive regulation of NF-kappaB transcription factor activity, positive regulation of NIK/NF-kappaB signaling, positive regulation of T-helper 1 cell cytokine production, signal transduction, T-helper 1 cell differentiation | murA2 | UDP-N-acetylglucosamine 1-carboxyvinyltransferase 2 | cytoplasm, UDP-N-acetylglucosamine 1-carboxyvinyltransferase activity, cell cycle, cell division, cell wall organization, peptidoglycan biosynthetic process, regulation of cell shape, UDP-N-acetylgalactosamine biosynthetic process |
| LRRFIP1 | J7MBD1 | Leucine-rich repeat flightless-interacting protein 1 | Transcriptional repressor which preferentially binds to the GC-rich consensus sequence (5'-AGCCCCCGGCG-3') and may regulate expression of TNF, EGFR and PDGFA. May control smooth muscle cells proliferation following artery injury through PDGFA repression. May also bind double-stranded RNA. Positively regulates Toll-like receptor (TLR) signaling in response to agonist probably by competing with the negative FLII regulator for MYD88-binding. | negative regulation of transcription by RNA polymerase II, positive regulation of NF-kappaB transcription factor activity, positive regulation of type I interferon production | M1GAS476_1767 | Cell surface protein | cell wall, extracellular region, integral component of membrane |
| LDHA | Q1CPG5 | L-lactate dehydrogenase A chain |  | glycolytic process, lactate metabolic process, NAD metabolic process, positive regulation of apoptotic process, post-embryonic animal organ development, pyruvate metabolic process, response to cAMP, response to drug, response to estrogen, response to glucose, response to hydrogen peroxide, response to hypoxia, response to nutrient, substantia nigra development | MGAS10750_Spy0594 | Phage protein | endonuclease activity, mRNA binding |
| PLIN2 | Q1J5S8 | Perilipin-2 | May be involved in development and maintenance of adipose tissue. | lipid storage, long-chain fatty acid transport, positive regulation of sequestering of triglyceride, regulation of lipid metabolic process, response to drug, response to organic cyclic compound | MGAS10750_Spy1357 | Uncharacterized protein |  |
| PLIN2 | Q1JDQ7 | Perilipin-2 | May be involved in development and maintenance of adipose tissue. | lipid storage, long-chain fatty acid transport, positive regulation of sequestering of triglyceride, regulation of lipid metabolic process, response to drug, response to organic cyclic compound |  |  |  |
| NRSN1 | H8HD54 | Neurensin-1 | May play an important role in neural organelle transport, and in transduction of nerve signals or in nerve growth. May play a role in neurite extension. May play a role in memory consolidation (By similarity). | nervous system development |  |  |  |
| LRRK1 | Q3K0D0 | Leucine-rich repeat serine/threonine-protein kinase 1 |  | bone resorption, negative regulation of peptidyl-tyrosine phosphorylation, osteoclast development, positive regulation of canonical Wnt signaling pathway, positive regulation of intracellular signal transduction, positive regulation of peptidyl-tyrosine phosphorylation, signal transduction | rplT | 50S ribosomal protein L20 | ribosome, rRNA binding, structural constituent of ribosome, ribosomal large subunit assembly, translation |
| EIF4A2 | Q490D8 | Eukaryotic initiation factor 4A-II | ATP-dependent RNA helicase which is a subunit of the eIF4F complex involved in cap recognition and is required for mRNA binding to ribosome. In the current model of translation initiation, eIF4A unwinds RNA secondary structures in the 5'-UTR of mRNAs which is necessary to allow efficient binding of the small ribosomal subunit, and subsequent scanning for the initiator codon. | cellular response to leukemia inhibitory factor, cytoplasmic translational initiation, negative regulation of RNA-directed 5'-3' RNA polymerase activity, regulation of translational initiation, translational initiation, viral process | rpmG1 | 50S ribosomal protein L33 1 | ribosome, structural constituent of ribosome, translation |
| LAPTM5 | Q1CPG5 | Lysosomal-associated transmembrane protein 5 | May have a special functional role during embryogenesis and in adult hematopoietic cells. | activation of cysteine-type endopeptidase activity involved in apoptotic process, cellular response to leukemia inhibitory factor, defense response to tumor cell, Golgi to lysosome transport, induction of programmed cell death, intracellular protein transport, negative regulation of activated T cell proliferation, negative regulation of autophagic cell death, negative regulation of B cell activation, negative regulation of interferon-gamma production, negative regulation of interleukin-2 production, negative regulation of T cell activation, negative regulation of T cell receptor signaling pathway, positive regulation of cytokine production involved in immune response, positive regulation of interleukin-12 production, positive regulation of interleukin-6 production, positive regulation of lysosomal membrane permeability, positive regulation of macrophage cytokine production, positive regulation of MAPK cascade, positive regulation of NIK/NF-kappaB signaling, positive regulation of protein ubiquitination, positive regulation of receptor catabolic process, positive regulation of tumor necrosis factor-mediated signaling pathway, positive regulation of ubiquitin-dependent protein catabolic process, protein targeting to lysosome | MGAS10750_Spy0594 | Phage protein | endonuclease activity, mRNA binding |
| HSP90B1 | H8HC58 | Endoplasmin | Molecular chaperone that functions in the processing and transport of secreted proteins (By similarity). When associated with CNPY3, required for proper folding of Toll-like receptors (By similarity). Functions in endoplasmic reticulum associated degradation (ERAD) (PubMed:18264092). Has ATPase activity (By similarity). May participate in the unfolding of cytosolic leaderless cargos (lacking the secretion signal sequence) such as the interleukin 1/IL-1 to facilitate their translocation into the ERGIC (endoplasmic reticulum-Golgi intermediate compartment) and secretion; the translocation process is mediated by the cargo receptor TMED10 (PubMed:32272059). | actin rod assembly, ATF6-mediated unfolded protein response, cellular protein metabolic process, cellular response to ATP, cytokine-mediated signaling pathway, negative regulation of apoptotic process, post-translational protein modification, protein folding, protein folding in endoplasmic reticulum, protein transport, receptor-mediated endocytosis, regulation of phosphoprotein phosphatase activity, response to endoplasmic reticulum stress, response to hypoxia, retrograde protein transport, ER to cytosol, sequestering of calcium ion, toll-like receptor signaling pathway, ubiquitin-dependent ERAD pathway |  |  |  |
| ZNF354B | K4N7Q1 | Zinc finger protein 354B | May be involved in transcriptional regulation. | negative regulation of transcription by RNA polymerase II, regulation of transcription by RNA polymerase II |  |  |  |
| ZNF354B | Q1J9L2 | Zinc finger protein 354B | May be involved in transcriptional regulation. | negative regulation of transcription by RNA polymerase II, regulation of transcription by RNA polymerase II |  |  |  |
| ZNF354B | Q1JK93 | Zinc finger protein 354B | May be involved in transcriptional regulation. | negative regulation of transcription by RNA polymerase II, regulation of transcription by RNA polymerase II | MGAS9429_Spy1542 | Uncharacterized protein |  |
| ZNF354B | Q878I9 | Zinc finger protein 354B | May be involved in transcriptional regulation. | negative regulation of transcription by RNA polymerase II, regulation of transcription by RNA polymerase II |  |  |  |
| ZNF354B | Q8K763 | Zinc finger protein 354B | May be involved in transcriptional regulation. | negative regulation of transcription by RNA polymerase II, regulation of transcription by RNA polymerase II |  |  |  |
| ZNF354B | Q99YH2 | Zinc finger protein 354B | May be involved in transcriptional regulation. | negative regulation of transcription by RNA polymerase II, regulation of transcription by RNA polymerase II | lacB1 | Galactose-6-phosphate isomerase subunit LacB 1 | galactose-6-phosphate isomerase activity, galactose catabolic process, lactose catabolic process via tagatose-6-phosphate |
| CD55 | Q99Z78 | Complement decay-accelerating factor | This protein recognizes C4b and C3b fragments that condense with cell-surface hydroxyl or amino groups when nascent C4b and C3b are locally generated during C4 and c3 activation. Interaction of daf with cell-associated C4b and C3b polypeptides interferes with their ability to catalyze the conversion of C2 and factor B to enzymatically active C2a and Bb and thereby prevents the formation of C4b2a and C3bBb, the amplification convertases of the complement cascade (PubMed:7525274). Inhibits complement activation by destabilizing and preventing the formation of C3 and C5 convertases, which prevents complement damage (PubMed:28657829). | CD4-positive, alpha-beta T cell cytokine production, complement activation, classical pathway, endoplasmic reticulum to Golgi vesicle-mediated transport, innate immune response, negative regulation of complement activation, neutrophil degranulation, positive regulation of CD4-positive, alpha-beta T cell activation, positive regulation of CD4-positive, alpha-beta T cell proliferation, positive regulation of cytosolic calcium ion concentration, regulation of complement activation, regulation of complement-dependent cytotoxicity, regulation of lipopolysaccharide-mediated signaling pathway, respiratory burst | murA2 | UDP-N-acetylglucosamine 1-carboxyvinyltransferase 2 | cytoplasm, UDP-N-acetylglucosamine 1-carboxyvinyltransferase activity, cell cycle, cell division, cell wall organization, peptidoglycan biosynthetic process, regulation of cell shape, UDP-N-acetylgalactosamine biosynthetic process |
| NAMPTP1 | B4U381 |  |  |  | Sez_1096 | Transposase |  |
| NAMPTP1 | Q1JND0 |  |  |  | MGAS9429_Spy0281 | Uncharacterized protein |  |

**Table S2: Interactions between human and *S.pyogenes* genes in polymicrobial infections and their corresponding protein functions.**

| **Human gene** | **Pathogen gene** | **Human full gene name** | **Human gene Function - from Uniprot** | **Function - from GO terms** | **Pathogen gene short name** | **Pathogen gene full name** | **Pathogen Function** |
| --- | --- | --- | --- | --- | --- | --- | --- |
| ZNF302 | A2REG3 | Zinc finger protein 302 | May be involved in transcriptional regulation. | regulation of transcription by RNA polymerase II |  |  |  |
| ZNF302 | H8HBC3 | Zinc finger protein 302 | May be involved in transcriptional regulation. | regulation of transcription by RNA polymerase II |  |  |  |
| ZNF302 | H8HC08 | Zinc finger protein 302 | May be involved in transcriptional regulation. | regulation of transcription by RNA polymerase II |  |  |  |
| ZNF302 | J7MBN2 | Zinc finger protein 302 | May be involved in transcriptional regulation. | regulation of transcription by RNA polymerase II | parB | Chromosome partitioning protein | DNA binding |
| ZNF302 | K4Q8H6 | Zinc finger protein 302 | May be involved in transcriptional regulation. | regulation of transcription by RNA polymerase II | ciaR | Response regulator mprA | DNA binding, phosphorelay signal transduction system, regulation of transcription, DNA-templated |
| ZNF302 | Q1J4A3 | Zinc finger protein 302 | May be involved in transcriptional regulation. | regulation of transcription by RNA polymerase II | MGAS10750_Spy1883 | NAD-dependent oxidoreductase | oxidoreductase activity |
| ZNF302 | Q1JP46 | Zinc finger protein 302 | May be involved in transcriptional regulation. | regulation of transcription by RNA polymerase II | MGAS9429_Spy0015 | Uncharacterized protein |  |
| ZNF302 | Q5XBG4 | Zinc finger protein 302 | May be involved in transcriptional regulation. | regulation of transcription by RNA polymerase II | nagB | Glucosamine-6-phosphate deaminase | glucosamine-6-phosphate deaminase activity, carbohydrate metabolic process, N-acetylglucosamine metabolic process, N-acetylneuraminate catabolic process |
| ZNF302 | Q7CFC6 | Zinc finger protein 302 | May be involved in transcriptional regulation. | regulation of transcription by RNA polymerase II |  |  |  |
| FKBP5 | Q5XE79 | Peptidyl-prolyl cis-trans isomerase FKBP5 | Immunophilin protein with PPIase and co-chaperone activities (PubMed:11350175). Component of unligated steroid receptors heterocomplexes through interaction with heat-shock protein 90 (HSP90). Plays a role in the intracellular trafficking of heterooligomeric forms of steroid hormone receptors maintaining the complex into the cytoplasm when unliganded (PubMed:12538866). Acts as a regulator of Akt/AKT1 activity by promoting the interaction between Akt/AKT1 and PHLPP1, thereby enhancing dephosphorylation and subsequent activation of Akt/AKT1 (PubMed:28147277). | chaperone-mediated protein folding, protein folding, protein peptidyl-prolyl isomerization, response to bacterium | M6_Spy0149 | Thioredoxin |  |
| PCSK5 | P58083 | Proprotein convertase subtilisin/kexin type 5 | Serine endoprotease that processes various proproteins by cleavage at paired basic amino acids, recognizing the RXXX[KR]R consensus motif. Likely functions in the constitutive and regulated secretory pathways. Plays an essential role in pregnancy establishment by proteolytic activation of a number of important factors such as BMP2, CALD1 and alpha-integrins. | anterior/posterior pattern specification, cell-cell signaling, cytokine precursor processing, embryo implantation, embryonic digestive tract development, embryonic skeletal system development, heart development, kidney development, limb morphogenesis, nerve growth factor processing, peptide biosynthetic process, peptide hormone processing, protein processing, regulation of lipoprotein lipase activity, renin secretion into blood stream, respiratory tube development, signal peptide processing, viral life cycle | hutH | Histidine ammonia-lyase | cytoplasm, histidine ammonia-lyase activity, histidine catabolic process to glutamate and formamide, histidine catabolic process to glutamate and formate |
| PCSK5 | Q1J4A3 | Proprotein convertase subtilisin/kexin type 5 | Serine endoprotease that processes various proproteins by cleavage at paired basic amino acids, recognizing the RXXX[KR]R consensus motif. Likely functions in the constitutive and regulated secretory pathways. Plays an essential role in pregnancy establishment by proteolytic activation of a number of important factors such as BMP2, CALD1 and alpha-integrins. | anterior/posterior pattern specification, cell-cell signaling, cytokine precursor processing, embryo implantation, embryonic digestive tract development, embryonic skeletal system development, heart development, kidney development, limb morphogenesis, nerve growth factor processing, peptide biosynthetic process, peptide hormone processing, protein processing, regulation of lipoprotein lipase activity, renin secretion into blood stream, respiratory tube development, signal peptide processing, viral life cycle | MGAS10750_Spy1883 | NAD-dependent oxidoreductase | oxidoreductase activity |
| PCSK5 | Q9A176 | Proprotein convertase subtilisin/kexin type 5 | Serine endoprotease that processes various proproteins by cleavage at paired basic amino acids, recognizing the RXXX[KR]R consensus motif. Likely functions in the constitutive and regulated secretory pathways. Plays an essential role in pregnancy establishment by proteolytic activation of a number of important factors such as BMP2, CALD1 and alpha-integrins. | anterior/posterior pattern specification, cell-cell signaling, cytokine precursor processing, embryo implantation, embryonic digestive tract development, embryonic skeletal system development, heart development, kidney development, limb morphogenesis, nerve growth factor processing, peptide biosynthetic process, peptide hormone processing, protein processing, regulation of lipoprotein lipase activity, renin secretion into blood stream, respiratory tube development, signal peptide processing, viral life cycle | nrdI | Protein NrdI | FMN binding, cellular protein modification process |
| STAG2 | J7M1N4 | Cohesin subunit SA-2 | Component of cohesin complex, a complex required for the cohesion of sister chromatids after DNA replication. The cohesin complex apparently forms a large proteinaceous ring within which sister chromatids can be trapped. At anaphase, the complex is cleaved and dissociates from chromatin, allowing sister chromatids to segregate. The cohesin complex may also play a role in spindle pole assembly during mitosis. | cell division, meiotic cell cycle, mitotic spindle assembly, sister chromatid cohesion | malX | Maltodextrin-binding protein | plasma membrane, carbohydrate transmembrane transporter activity |
| ZPR1 | J7M1N4 | Zinc finger protein ZPR1 | Acts as a signaling molecule that communicates proliferative growth signals from the cytoplasm to the nucleus. Plays a role for the localization and accumulation of the survival motor neuron protein SMN1 in sub-nuclear bodies, including gems and Cajal bodies. Induces neuron differentiation and stimulates axonal growth and formation of growth cone in spinal cord motor neurons. Plays a role in the splicing of cellular pre-mRNAs. May be involved in H(2)O(2)-induced neuronal cell death. | apoptotic process involved in development, axon development, Cajal body organization, cell population proliferation, cellular response to epidermal growth factor stimulus, DNA endoreduplication, inner cell mass cell proliferation, microtubule cytoskeleton organization, mRNA processing, negative regulation of motor neuron apoptotic process, positive regulation of gene expression, positive regulation of growth, positive regulation of protein import into nucleus, positive regulation of RNA splicing, positive regulation of transcription involved in G1/S transition of mitotic cell cycle, pre-mRNA catabolic process, regulation of myelination, RNA splicing, signal transduction, spinal cord development, trophectodermal cell proliferation | malX | Maltodextrin-binding protein | plasma membrane, carbohydrate transmembrane transporter activity |
| ZPR1 | J7M850 | Zinc finger protein ZPR1 | Acts as a signaling molecule that communicates proliferative growth signals from the cytoplasm to the nucleus. Plays a role for the localization and accumulation of the survival motor neuron protein SMN1 in sub-nuclear bodies, including gems and Cajal bodies. Induces neuron differentiation and stimulates axonal growth and formation of growth cone in spinal cord motor neurons. Plays a role in the splicing of cellular pre-mRNAs. May be involved in H(2)O(2)-induced neuronal cell death. | apoptotic process involved in development, axon development, Cajal body organization, cell population proliferation, cellular response to epidermal growth factor stimulus, DNA endoreduplication, inner cell mass cell proliferation, microtubule cytoskeleton organization, mRNA processing, negative regulation of motor neuron apoptotic process, positive regulation of gene expression, positive regulation of growth, positive regulation of protein import into nucleus, positive regulation of RNA splicing, positive regulation of transcription involved in G1/S transition of mitotic cell cycle, pre-mRNA catabolic process, regulation of myelination, RNA splicing, signal transduction, spinal cord development, trophectodermal cell proliferation | M1GAS476_1200 | Competence protein/transcription factor |  |
| ZPR1 | Q1J4A3 | Zinc finger protein ZPR1 | Acts as a signaling molecule that communicates proliferative growth signals from the cytoplasm to the nucleus. Plays a role for the localization and accumulation of the survival motor neuron protein SMN1 in sub-nuclear bodies, including gems and Cajal bodies. Induces neuron differentiation and stimulates axonal growth and formation of growth cone in spinal cord motor neurons. Plays a role in the splicing of cellular pre-mRNAs. May be involved in H(2)O(2)-induced neuronal cell death. | apoptotic process involved in development, axon development, Cajal body organization, cell population proliferation, cellular response to epidermal growth factor stimulus, DNA endoreduplication, inner cell mass cell proliferation, microtubule cytoskeleton organization, mRNA processing, negative regulation of motor neuron apoptotic process, positive regulation of gene expression, positive regulation of growth, positive regulation of protein import into nucleus, positive regulation of RNA splicing, positive regulation of transcription involved in G1/S transition of mitotic cell cycle, pre-mRNA catabolic process, regulation of myelination, RNA splicing, signal transduction, spinal cord development, trophectodermal cell proliferation | MGAS10750_Spy1883 | NAD-dependent oxidoreductase | oxidoreductase activity |
| ZPR1 | Q3K0C8 | Zinc finger protein ZPR1 | Acts as a signaling molecule that communicates proliferative growth signals from the cytoplasm to the nucleus. Plays a role for the localization and accumulation of the survival motor neuron protein SMN1 in sub-nuclear bodies, including gems and Cajal bodies. Induces neuron differentiation and stimulates axonal growth and formation of growth cone in spinal cord motor neurons. Plays a role in the splicing of cellular pre-mRNAs. May be involved in H(2)O(2)-induced neuronal cell death. | apoptotic process involved in development, axon development, Cajal body organization, cell population proliferation, cellular response to epidermal growth factor stimulus, DNA endoreduplication, inner cell mass cell proliferation, microtubule cytoskeleton organization, mRNA processing, negative regulation of motor neuron apoptotic process, positive regulation of gene expression, positive regulation of growth, positive regulation of protein import into nucleus, positive regulation of RNA splicing, positive regulation of transcription involved in G1/S transition of mitotic cell cycle, pre-mRNA catabolic process, regulation of myelination, RNA splicing, signal transduction, spinal cord development, trophectodermal cell proliferation | infC | Translation initiation factor IF-3 | cytoplasm, translation initiation factor activity |
| SIAE | H8H8L5 | Sialate O-acetylesterase | Catalyzes the removal of O-acetyl ester groups from position 9 of the parent sialic acid, N-acetylneuraminic acid. | carbohydrate metabolic process, regulation of immune system process |  |  |  |
| SIAE | Q8E7Q8 | Sialate O-acetylesterase | Catalyzes the removal of O-acetyl ester groups from position 9 of the parent sialic acid, N-acetylneuraminic acid. | carbohydrate metabolic process, regulation of immune system process | grpE | Protein GrpE | cytoplasm, adenyl-nucleotide exchange factor activity, chaperone binding, protein homodimerization activity, protein folding |
| STAT1 | Q1JC89 | Signal transducer and activator of transcription 1-alpha/beta | Signal transducer and transcription activator that mediates cellular responses to interferons (IFNs), cytokine KITLG/SCF and other cytokines and other growth factors. Following type I IFN (IFN-alpha and IFN-beta) binding to cell surface receptors, signaling via protein kinases leads to activation of Jak kinases (TYK2 and JAK1) and to tyrosine phosphorylation of STAT1 and STAT2. The phosphorylated STATs dimerize and associate with ISGF3G/IRF-9 to form a complex termed ISGF3 transcription factor, that enters the nucleus (PubMed:28753426). ISGF3 binds to the IFN stimulated response element (ISRE) to activate the transcription of IFN-stimulated genes (ISG), which drive the cell in an antiviral state. In response to type II IFN (IFN-gamma), STAT1 is tyrosine- and serine-phosphorylated (PubMed:26479788). It then forms a homodimer termed IFN-gamma-activated factor (GAF), migrates into the nucleus and binds to the IFN gamma activated sequence (GAS) to drive the expression of the target genes, inducing a cellular antiviral state. Becomes activated in response to KITLG/SCF and KIT signaling. May mediate cellular responses to activated FGFR1, FGFR2, FGFR3 and FGFR4. | blood circulation, cellular response to interferon-beta, cellular response to interferon-gamma, cytokine-mediated signaling pathway, defense response, defense response to virus, interferon-gamma-mediated signaling pathway, interleukin-21-mediated signaling pathway, interleukin-27-mediated signaling pathway, interleukin-35-mediated signaling pathway, interleukin-6-mediated signaling pathway, interleukin-9-mediated signaling pathway, macrophage derived foam cell differentiation, metanephric mesenchymal cell differentiation, metanephric mesenchymal cell proliferation involved in metanephros development, negative regulation by virus of viral protein levels in host cell, negative regulation of angiogenesis, negative regulation of endothelial cell proliferation, negative regulation of I-kappaB kinase/NF-kappaB signaling, negative regulation of mesenchymal to epithelial transition involved in metanephros morphogenesis, negative regulation of metanephric nephron tubule epithelial cell differentiation, negative regulation of transcription by RNA polymerase II, positive regulation of defense response to virus by host, positive regulation of erythrocyte differentiation, positive regulation of interferon-alpha production, positive regulation of mesenchymal cell proliferation, positive regulation of smooth muscle cell proliferation, positive regulation of transcription by RNA polymerase II, positive regulation of transcription of Notch receptor target, positive regulation of transcription, DNA-templated, receptor signaling pathway via JAK-STAT, regulation of apoptotic process, regulation of cell population proliferation, regulation of interferon-gamma-mediated signaling pathway, regulation of transcription, DNA-templated, renal tubule development, response to cAMP, response to cytokine, response to interferon-beta, response to peptide hormone, tumor necrosis factor-mediated signaling pathway, type I interferon signaling pathway, viral process | rpiA | Ribose-5-phosphate isomerase A | ribose-5-phosphate isomerase activity, pentose-phosphate shunt, non-oxidative branch |
| IL18R1 | P60381 | Interleukin-18 receptor 1 | Within the IL18 receptor complex, responsible for the binding of the proinflammatory cytokine IL18, but not IL1A nor IL1B (PubMed:8626725, PubMed:14528293, PubMed:25261253, PubMed:25500532). Involved in IL18-mediated IFNG synthesis from T-helper 1 (Th1) cells (PubMed:10653850). Contributes to IL18-induced cytokine production, either independently of SLC12A3, or as a complex with SLC12A3 (By similarity). | cellular response to cytokine stimulus, immune response, inflammatory response, interleukin-18-mediated signaling pathway, natural killer cell activation, negative regulation of cold-induced thermogenesis, positive regulation of interferon-gamma production, positive regulation of NF-kappaB transcription factor activity, positive regulation of NIK/NF-kappaB signaling, positive regulation of T-helper 1 cell cytokine production, signal transduction, T-helper 1 cell differentiation | spx | Global transcriptional regulator Spx | cytoplasm, negative regulation of transcription, DNA-templated |
| KYNU | M4YYR0 | Kynureninase | Catalyzes the cleavage of L-kynurenine (L-Kyn) and L-3-hydroxykynurenine (L-3OHKyn) into anthranilic acid (AA) and 3-hydroxyanthranilic acid (3-OHAA), respectively. Has a preference for the L-3-hydroxy form. Also has cysteine-conjugate-beta-lyase activity. | 'de novo' NAD biosynthetic process from tryptophan, aging, anthranilate metabolic process, L-kynurenine catabolic process, NAD biosynthetic process, quinolinate biosynthetic process, response to interferon-gamma, response to vitamin B6, tryptophan catabolic process, tryptophan catabolic process to acetyl-CoA, tryptophan catabolic process to kynurenine | GGS_1650 | Phosphoglycerate mutase | isomerase activity |
| SFPQ | Q7CFC6 | Splicing factor, proline- and glutamine-rich | DNA- and RNA binding protein, involved in several nuclear processes. Essential pre-mRNA splicing factor required early in spliceosome formation and for splicing catalytic step II, probably as a heteromer with NONO. Binds to pre-mRNA in spliceosome C complex, and specifically binds to intronic polypyrimidine tracts. Involved in regulation of signal-induced alternative splicing. During splicing of PTPRC/CD45, a phosphorylated form is sequestered by THRAP3 from the pre-mRNA in resting T-cells; T-cell activation and subsequent reduced phosphorylation is proposed to lead to release from THRAP3 allowing binding to pre-mRNA splicing regulatotry elements which represses exon inclusion. Interacts with U5 snRNA, probably by binding to a purine-rich sequence located on the 3' side of U5 snRNA stem 1b. May be involved in a pre-mRNA coupled splicing and polyadenylation process as component of a snRNP-free complex with SNRPA/U1A. The SFPQ-NONO heteromer associated with MATR3 may play a role in nuclear retention of defective RNAs. SFPQ may be involved in homologous DNA pairing; in vitro, promotes the invasion of ssDNA between a duplex DNA and produces a D-loop formation. The SFPQ-NONO heteromer may be involved in DNA unwinding by modulating the function of topoisomerase I/TOP1; in vitro, stimulates dissociation of TOP1 from DNA after cleavage and enhances its jumping between separate DNA helices. The SFPQ-NONO heteromer binds DNA (PubMed:25765647). The SFPQ-NONO heteromer may be involved in DNA non-homologous end joining (NHEJ) required for double-strand break repair and V(D)J recombination and may stabilize paired DNA ends; in vitro, the complex strongly stimulates DNA end joining, binds directly to the DNA substrates and cooperates with the Ku70/G22P1-Ku80/XRCC5 (Ku) dimer to establish a functional preligation complex. SFPQ is involved in transcriptional regulation. Functions as transcriptional activator (PubMed:25765647). Transcriptional repression is mediated by an interaction of SFPQ with SIN3A and subsequent recruitment of histone deacetylases (HDACs). The SFPQ-NONO-NR5A1 complex binds to the CYP17 promoter and regulates basal and cAMP-dependent transcriptional activity. SFPQ isoform Long binds to the DNA binding domains (DBD) of nuclear hormone receptors, like RXRA and probably THRA, and acts as transcriptional corepressor in absence of hormone ligands. Binds the DNA sequence 5'-CTGAGTC-3' in the insulin-like growth factor response element (IGFRE) and inhibits IGF-I-stimulated transcriptional activity. Regulates the circadian clock by repressing the transcriptional activator activity of the CLOCK-ARNTL/BMAL1 heterodimer. Required for the transcriptional repression of circadian target genes, such as PER1, mediated by the large PER complex through histone deacetylation (By similarity). Required for the assembly of nuclear speckles (PubMed:25765647). Plays a role in the regulation of DNA virus-mediated innate immune response by assembling into the HDP-RNP complex, a complex that serves as a platform for IRF3 phosphorylation and subsequent innate immune response activation through the cGAS-STING pathway (PubMed:28712728). | activation of innate immune response, alternative mRNA splicing, via spliceosome, dendritic transport of messenger ribonucleoprotein complex, double-strand break repair via homologous recombination, histone H3 deacetylation, innate immune response, mRNA processing, mRNA splicing, via spliceosome, negative regulation of circadian rhythm, negative regulation of transcription by RNA polymerase II, negative regulation of transcription, DNA-templated, positive regulation of oxidative stress-induced intrinsic apoptotic signaling pathway, positive regulation of sister chromatid cohesion, positive regulation of transcription by RNA polymerase II, regulation of circadian rhythm, regulation of transcription, DNA-templated, rhythmic process, RNA splicing |  |  |  |
| POSTN | G4R4C9 | Periostin | Induces cell attachment and spreading and plays a role in cell adhesion (PubMed:12235007). Enhances incorporation of BMP1 in the fibronectin matrix of connective tissues, and subsequent proteolytic activation of lysyl oxidase LOX (By similarity). | bone regeneration, cell adhesion, cellular response to fibroblast growth factor stimulus, cellular response to transforming growth factor beta stimulus, cellular response to tumor necrosis factor, cellular response to vitamin K, extracellular matrix organization, negative regulation of cell-matrix adhesion, negative regulation of substrate adhesion-dependent cell spreading, neuron projection extension, positive regulation of chemokine (C-X-C motif) ligand 2 production, positive regulation of smooth muscle cell migration, regulation of Notch signaling pathway, regulation of systemic arterial blood pressure, response to estradiol, response to hypoxia, response to mechanical stimulus, response to muscle activity, wound healing |  |  |  |
| CXCL9 | B5XIX4 | C-X-C motif chemokine 9 | Cytokine that affects the growth, movement, or activation state of cells that participate in immune and inflammatory response. Chemotactic for activated T-cells. Binds to CXCR3. | adenylate cyclase-activating G protein-coupled receptor signaling pathway, antimicrobial humoral immune response mediated by antimicrobial peptide, cell-cell signaling, cellular defense response, cellular response to lipopolysaccharide, chemokine-mediated signaling pathway, chemotaxis, defense response, defense response to virus, G protein-coupled receptor signaling pathway, immune response, inflammatory response, killing of cells of other organism, leukocyte chemotaxis, neutrophil chemotaxis, positive regulation of myoblast differentiation, positive regulation of myoblast fusion, positive regulation of release of sequestered calcium ion into cytosol, regulation of cell population proliferation, signal transduction |  |  |  |
| CXCL5 | P60381 | C-X-C motif chemokine 5 | Involved in neutrophil activation. In vitro, ENA-78(8-78) and ENA-78(9-78) show a threefold higher chemotactic activity for neutrophil granulocytes. | antimicrobial humoral immune response mediated by antimicrobial peptide, cell-cell signaling, cellular response to lipopolysaccharide, chemokine-mediated signaling pathway, chemotaxis, G protein-coupled receptor signaling pathway, immune response, inflammatory response, leukocyte chemotaxis, neutrophil chemotaxis, positive regulation of cell population proliferation, signal transduction | spx | Global transcriptional regulator Spx | cytoplasm, negative regulation of transcription, DNA-templated |
| TAF1D | P63443 | TATA box-binding protein-associated factor RNA polymerase I subunit D | Component of the transcription factor SL1/TIF-IB complex, which is involved in the assembly of the PIC (preinitiation complex) during RNA polymerase I-dependent transcription. The rate of PIC formation probably is primarily dependent on the rate of association of SL1/TIF-IB with the rDNA promoter. SL1/TIF-IB is involved in stabilization of nucleolar transcription factor 1/UBTF on rDNA. Formation of SL1/TIF-IB excludes the association of TBP with TFIID subunits. | positive regulation of gene expression, epigenetic, regulation of transcription, DNA-templated, termination of RNA polymerase I transcription, transcription elongation from RNA polymerase I promoter, transcription initiation from RNA polymerase I promoter | acpP | Acyl carrier protein | cytoplasm, acyl carrier activity |
| GOLGB1 | Q1JE27 | Golgin subfamily B member 1 | May participate in forming intercisternal cross-bridges of the Golgi complex. | endoplasmic reticulum to Golgi vesicle-mediated transport, Golgi organization, protein localization to pericentriolar material, regulation of transcription, DNA-templated |  |  |  |
| CHD2 | P68893 | Chromodomain-helicase-DNA-binding protein 2 | DNA-binding helicase that specifically binds to the promoter of target genes, leading to chromatin remodeling, possibly by promoting deposition of histone H3.3. Involved in myogenesis via interaction with MYOD1: binds to myogenic gene regulatory sequences and mediates incorporation of histone H3.3 prior to the onset of myogenic gene expression, promoting their expression (By similarity). | chromatin organization, muscle organ development, regulation of transcription by RNA polymerase II | nusG | Transcription termination/antitermination protein NusG | DNA-templated transcription, elongation, DNA-templated transcription, termination, regulation of DNA-templated transcription, elongation, transcription antitermination |
| ZNF354B | H8H8L9 | Zinc finger protein 354B | May be involved in transcriptional regulation. | negative regulation of transcription by RNA polymerase II, regulation of transcription by RNA polymerase II |  |  |  |
| A2RFD6 | A2RFD6 |  |  |  |  |  |  |
| A2RFD6 | G4R1I5 |  |  |  |  |  |  |
| FTH1P2 | G4R415 |  |  |  |  |  |  |
| FTH1P2 | H8HFZ6 |  |  |  |  |  |  |
| FTH1P2 | Q1JHQ0 |  |  |  | sagF | Streptolysin S biosynthesis protein SagF | integral component of membrane |
| FTH1P2 | Q3K010 |  |  |  | pyrH | Uridylate kinase | cytoplasm, ATP binding, UMP kinase activity, 'de novo' CTP biosynthetic process |
| FTH1P2 | Q3K3U3 |  |  |  | rplQ | 50S ribosomal protein L17 | ribosome, structural constituent of ribosome, translation |
| FTH1P2 | Q8K7H9 |  |  |  |  |  |  |
| FTH1P2 | Q9ZHG8 |  |  |  | lmb | Lmb | metal ion binding, cell adhesion, metal ion transport |
| TXNIP | C5WJM1 | Thioredoxin-interacting protein | May act as an oxidative stress mediator by inhibiting thioredoxin activity or by limiting its bioavailability. Interacts with COPS5 and restores COPS5-induced suppression of CDKN1B stability, blocking the COPS5-mediated translocation of CDKN1B from the nucleus to the cytoplasm. Functions as a transcriptional repressor, possibly by acting as a bridge molecule between transcription factors and corepressor complexes, and over-expression will induce G0/G1 cell cycle arrest. Required for the maturation of natural killer cells. Acts as a suppressor of tumor cell growth. Inhibits the proteasomal degradation of DDIT4, and thereby contributes to the inhibition of the mammalian target of rapamycin complex 1 (mTORC1). | cell cycle, cellular response to tumor cell, keratinocyte differentiation, negative regulation of cell division, negative regulation of transcription by RNA polymerase II, platelet-derived growth factor receptor signaling pathway, positive regulation of apoptotic process, protein import into nucleus, protein transport, regulation of cell population proliferation, response to calcium ion, response to drug, response to estradiol, response to glucose, response to hydrogen peroxide, response to mechanical stimulus, response to progesterone | SDEG_2107 | Uncharacterized protein | integral component of membrane |

**Table S3: Interactions between human and *P.asaccharolytica* genes in polymicrobial infections and their corresponding protein functions.**

| **Human gene** | **Pathogen gene** | **Human full gene name** | **Human gene function - from Uniprot** | **Function - from GO terms** | **Pathogen gene short name** | **Pathogen gene full name** | **Pathogen Function** |
| --- | --- | --- | --- | --- | --- | --- | --- |
| SLC11A1 | F4KMQ7 | Natural resistance-associated macrophage protein 1 | Divalent transition metal (iron and manganese) transporter involved in iron metabolism and host resistance to certain pathogens. Macrophage-specific membrane transport function. Controls natural resistance to infection with intracellular parasites. Pathogen resistance involves sequestration of Fe(2+) and Mn(2+), cofactors of both prokaryotic and eukaryotic catalases and superoxide dismutases, not only to protect the macrophage against its own generation of reactive oxygen species, but to deny the cations to the pathogen for synthesis of its protective enzymes. | activation of protein kinase activity, antigen processing and presentation of peptide antigen, antimicrobial humoral response, cadmium ion transmembrane transport, cell redox homeostasis, cellular cadmium ion homeostasis, cellular iron ion homeostasis, defense response to bacterium, defense response to Gram-negative bacterium, defense response to protozoan, divalent metal ion export, inflammatory response, interleukin-2 production, interleukin-3 production, iron ion homeostasis, iron ion transport, L-arginine transport, macrophage activation, manganese ion transport, MHC class II biosynthetic process, mRNA stabilization, multicellular organismal iron ion homeostasis, negative regulation of cytokine production, neutrophil degranulation, nitrite transport, phagocytosis, positive regulation of cytokine production, positive regulation of dendritic cell antigen processing and presentation, positive regulation of gene expression, positive regulation of interferon-gamma production, positive regulation of phagocytosis, positive regulation of T-helper 1 type immune response, positive regulation of transcription by RNA polymerase II, respiratory burst, response to bacterium, response to interferon-gamma, response to lipopolysaccharide, T cell cytokine production, T cell proliferation involved in immune response, vacuolar acidification, wound healing | rplF | 50S ribosomal protein L6 | ribosome, rRNA binding, structural constituent of ribosome, translation |
| SIAE | F4KJP1 | Sialate O-acetylesterase | Catalyzes the removal of O-acetyl ester groups from position 9 of the parent sialic acid, N-acetylneuraminic acid. | carbohydrate metabolic process, regulation of immune system process | rpmF | 50S ribosomal protein L32 | large ribosomal subunit, structural constituent of ribosome, translation |
| SIAE | F4KK28 |  |  |  | Poras_0808 | Heat shock protein Hsp20 |  |
| SIAE | F4KKZ1 |  |  |  | rplU | 50S ribosomal protein L21 | ribosome, rRNA binding, structural constituent of ribosome, translation |
| HINT3 | F4KK28 | Histidine triad nucleotide-binding protein 3 | Hydrolyzes phosphoramidate and acyl-adenylate substrates. |  | Poras_0808 | Heat shock protein Hsp20 |  |
| HINT3 | F4KKZ1 |  |  |  | rplU | 50S ribosomal protein L21 | ribosome, rRNA binding, structural constituent of ribosome, translation |
| HINT3 | F4KMQ7 |  |  |  | rplF | 50S ribosomal protein L6 | ribosome, rRNA binding, structural constituent of ribosome, translation |
| KYNU | F4KJP1 | Kynureninase | Catalyzes the cleavage of L-kynurenine (L-Kyn) and L-3-hydroxykynurenine (L-3OHKyn) into anthranilic acid (AA) and 3-hydroxyanthranilic acid (3-OHAA), respectively. Has a preference for the L-3-hydroxy form. Also has cysteine-conjugate-beta-lyase activity. | de novo' NAD biosynthetic process from tryptophan, aging, anthranilate metabolic process, L-kynurenine catabolic process, NAD biosynthetic process, quinolinate biosynthetic process, response to interferon-gamma, response to vitamin B6, tryptophan catabolic process, tryptophan catabolic process to acetyl-CoA, tryptophan catabolic process to kynurenine | rpmF | 50S ribosomal protein L32 | large ribosomal subunit, structural constituent of ribosome, translation |
| KYNU | F4KK28 |  |  |  | Poras_0808 | Heat shock protein Hsp20 |  |
| KYNU | F4KKZ1 |  |  |  | rplU | 50S ribosomal protein L21 | ribosome, rRNA binding, structural constituent of ribosome, translation |
| KYNU | F4KM34 |  |  |  | rpsR | 30S ribosomal protein S18 | ribosome, rRNA binding, structural constituent of ribosome, translation |
| KYNU | F4KMQ7 |  |  |  | rplF | 50S ribosomal protein L6 | ribosome, rRNA binding, structural constituent of ribosome, translation |
| KYNU | F4KMS5 |  |  |  | rpsG | 30S ribosomal protein S7 | small ribosomal subunit, rRNA binding, structural constituent of ribosome, tRNA binding, translation |
| TGFBI | F4KKZ1 | Transforming growth factor-beta-induced protein ig-h3 | Plays a role in cell adhesion (PubMed:8024701). May play a role in cell-collagen interactions (By similarity). | angiogenesis, cell adhesion, cell population proliferation, cellular protein metabolic process, chondrocyte differentiation, extracellular matrix organization, negative regulation of cell adhesion, response to stimulus, visual perception | rplU | 50S ribosomal protein L21 | ribosome, rRNA binding, structural constituent of ribosome, translation |
| TGFBI | F4KLX1 |  |  |  | Poras_0205 | RNA polymerase, sigma-24 subunit, ECF subfamily | DNA binding, DNA-binding transcription factor activity, sigma factor activity, DNA-templated transcription, initiation |
| TGFBI | F4KM34 |  |  |  | rpsR | 30S ribosomal protein S18 | ribosome, rRNA binding, structural constituent of ribosome, translation |
| TGFBI | F4KMD3 |  |  |  | rpsO | 30S ribosomal protein S15 | ribosome, rRNA binding, structural constituent of ribosome, translation |
| TGFBI | F4KMQ7 |  |  |  | rplF | 50S ribosomal protein L6 | ribosome, rRNA binding, structural constituent of ribosome, translation |
| TGFBI | F4KMS5 |  |  |  | rpsG | 30S ribosomal protein S7 | small ribosomal subunit, rRNA binding, structural constituent of ribosome, tRNA binding, translation |
| TGFBI | F4KP35 |  |  |  | Poras_1633 | Protease Do | aminopeptidase activity, serine-type endopeptidase activity |
| LCP1 | F4KK28 | Plastin-2 | Actin-binding protein (PubMed:16636079, PubMed:17294403, PubMed:28493397). Plays a role in the activation of T-cells in response to costimulation through TCR/CD3 and CD2 or CD28 (PubMed:17294403). Modulates the cell surface expression of IL2RA/CD25 and CD69 (PubMed:17294403). | actin filament bundle assembly, actin filament network formation, animal organ regeneration, cell migration, extracellular matrix disassembly, interleukin-12-mediated signaling pathway, positive regulation of podosome assembly, protein kinase A signaling, regulation of intracellular protein transport, T cell activation involved in immune response | Poras_0808 | Heat shock protein Hsp20 |  |
| BCL2A1 | F4KK28 | Bcl-2-related protein A1 | Retards apoptosis induced by IL-3 deprivation. May function in the response of hemopoietic cells to external signals and in maintaining endothelial survival during infection (By similarity). Can inhibit apoptosis induced by serum starvation in the mammary epithelial cell line HC11 (By similarity). | aging, cerebral cortex development, extrinsic apoptotic signaling pathway in absence of ligand, intrinsic apoptotic signaling pathway in response to DNA damage, negative regulation of apoptotic process | Poras_0808 | Heat shock protein Hsp20 |  |
| ANPEP | F4KMQ7 | Aminopeptidase N | Broad specificity aminopeptidase which plays a role in the final digestion of peptides generated from hydrolysis of proteins by gastric and pancreatic proteases. Also involved in the processing of various peptides including peptide hormones, such as angiotensin III and IV, neuropeptides, and chemokines. May also be involved the cleavage of peptides bound to major histocompatibility complex class II molecules of antigen presenting cells. May have a role in angiogenesis and promote cholesterol crystallization. May have a role in amino acid transport by acting as binding partner of amino acid transporter SLC6A19 and regulating its activity (By similarity). | angiogenesis, cell differentiation, neutrophil degranulation, peptide catabolic process, proteolysis, regulation of blood pressure, signal transduction | rplF | 50S ribosomal protein L6 | ribosome, rRNA binding, structural constituent of ribosome, translation |
| AKAP13 | F4KK28 | A-kinase anchor protein 13 | Scaffold protein that plays an important role in assembling signaling complexes downstream of several types of G protein-coupled receptors. Activates RHOA in response to signaling via G protein-coupled receptors via its function as Rho guanine nucleotide exchange factor (PubMed:11546812, PubMed:15229649, PubMed:23090968, PubMed:25186459, PubMed:24993829). May also activate other Rho family members (PubMed:11546812). Part of a kinase signaling complex that links ADRA1A and ADRA1B adrenergic receptor signaling to the activation of downstream p38 MAP kinases, such as MAPK11 and MAPK14 (PubMed:17537920, PubMed:23716597, PubMed:21224381). Part of a signaling complex that links ADRA1B signaling to the activation of RHOA and IKBKB/IKKB, leading to increased NF-kappa-B transcriptional activity (PubMed:23090968). Part of a RHOA-dependent signaling cascade that mediates responses to lysophosphatidic acid (LPA), a signaling molecule that activates G-protein coupled receptors and potentiates transcriptional activation of the glucocorticoid receptor NR3C1 (PubMed:16469733). Part of a signaling cascade that stimulates MEF2C-dependent gene expression in response to lysophosphatidic acid (LPA) (By similarity). Part of a signaling pathway that activates MAPK11 and/or MAPK14 and leads to increased transcription activation of the estrogen receptors ESR1 and ESR2 (PubMed:9627117, PubMed:11579095). Part of a signaling cascade that links cAMP and EGFR signaling to BRAF signaling and to PKA-mediated phosphorylation of KSR1, leading to the activation of downstream MAP kinases, such as MAPK1 or MAPK3 (PubMed:21102438). Functions as scaffold protein that anchors cAMP-dependent protein kinase (PKA) and PRKD1. This promotes activation of PRKD1, leading to increased phosphorylation of HDAC5 and ultimately cardiomyocyte hypertrophy (By similarity). Has no guanine nucleotide exchange activity on CDC42, Ras or Rac (PubMed:11546812). Required for normal embryonic heart development, and in particular for normal sarcomere formation in the developing cardiomyocytes (By similarity). Plays a role in cardiomyocyte growth and cardiac hypertrophy in response to activation of the beta-adrenergic receptor by phenylephrine or isoproterenol (PubMed:17537920, PubMed:23090968). Required for normal adaptive cardiac hypertrophy in response to pressure overload (PubMed:23716597). Plays a role in osteogenesis (By similarity). | adenylate cyclase-activating adrenergic receptor signaling pathway involved in heart process, adrenergic receptor signaling pathway, bone development, cardiac muscle cell differentiation, cell growth involved in cardiac muscle cell development, G protein-coupled receptor signaling pathway, heart development, nuclear export, positive regulation of apoptotic process, positive regulation of I-kappaB kinase/NF-kappaB signaling, positive regulation of MAP kinase activity, positive regulation of Rho protein signal transduction, regulation of glucocorticoid mediated signaling pathway, regulation of Rho protein signal transduction, regulation of sarcomere organization, regulation of small GTPase mediated signal transduction | Poras_0808 | Heat shock protein Hsp20 |  |
| CD163 | F4KMS5 | Scavenger receptor cysteine-rich type 1 protein M130 | Acute phase-regulated receptor involved in clearance and endocytosis of hemoglobin/haptoglobin complexes by macrophages and may thereby protect tissues from free hemoglobin-mediated oxidative damage. May play a role in the uptake and recycling of iron, via endocytosis of hemoglobin/haptoglobin and subsequent breakdown of heme. Binds hemoglobin/haptoglobin complexes in a calcium-dependent and pH-dependent manner. Exhibits a higher affinity for complexes of hemoglobin and multimeric haptoglobin of HP*1F phenotype than for complexes of hemoglobin and dimeric haptoglobin of HP*1S phenotype. Induces a cascade of intracellular signals that involves tyrosine kinase-dependent calcium mobilization, inositol triphosphate production and secretion of IL6 and CSF1. Isoform 3 exhibits the higher capacity for ligand endocytosis and the more pronounced surface expression when expressed in cells. | acute-phase response, receptor-mediated endocytosis | rpsG | 30S ribosomal protein S7 | small ribosomal subunit, rRNA binding, structural constituent of ribosome, tRNA binding, translation |
| RALGAPA2 | F4KLX1 | Ral GTPase-activating protein subunit alpha-2 | Catalytic subunit of the heterodimeric RalGAP2 complex which acts as a GTPase activator for the Ras-like small GTPases RALA and RALB. | activation of GTPase activity, regulation of small GTPase mediated signal transduction | Poras_0205 | RNA polymerase, sigma-24 subunit, ECF subfamily | DNA binding, DNA-binding transcription factor activity, sigma factor activity, DNA-templated transcription, initiation |
| RALGAPA2 | F4KP35 |  |  |  | Poras_1633 | Protease Do | aminopeptidase activity, serine-type endopeptidase activity |
| PPIA | F4KM34 | Peptidyl-prolyl cis-trans isomerase A | Catalyzes the cis-trans isomerization of proline imidic peptide bonds in oligopeptides (PubMed:2001362, PubMed:20676357, PubMed:21245143, PubMed:25678563, PubMed:21593166). Exerts a strong chemotactic effect on leukocytes partly through activation of one of its membrane receptors BSG/CD147, initiating a signaling cascade that culminates in MAPK/ERK activation (PubMed:11943775, PubMed:21245143). Activates endothelial cells (ECs) in a proinflammatory manner by stimulating activation of NF-kappa-B and ERK, JNK and p38 MAP-kinases and by inducing expression of adhesion molecules including SELE and VCAM1 (PubMed:15130913). Induces apoptosis in ECs by promoting the FOXO1-dependent expression of CCL2 and BCL2L11 which are involved in EC chemotaxis and apoptosis (PubMed:31063815). In response to oxidative stress, initiates proapoptotic and antiapoptotic signaling in ECs via activation of NF-kappa-B and AKT1 and up-regulation of antiapoptotic protein BCL2 (PubMed:23180369). Negatively regulates MAP3K5/ASK1 kinase activity, autophosphorylation and oxidative stress-induced apoptosis mediated by MAP3K5/ASK1 (PubMed:26095851). Necessary for the assembly of TARDBP in heterogeneous nuclear ribonucleoprotein (hnRNP) complexes and regulates TARDBP binding to RNA UG repeats and TARDBP-dependent expression of HDAC6, ATG7 and VCP which are involved in clearance of protein aggregates (PubMed:25678563). Plays an important role in platelet activation and aggregation (By similarity). Regulates calcium mobilization and integrin ITGA2B:ITGB3 bidirectional signaling via increased ROS production as well as by facilitating the interaction between integrin and the cell cytoskeleton (By similarity). Binds heparan sulfate glycosaminoglycans (PubMed:11943775). Inhibits replication of influenza A virus (IAV) (PubMed:19207730). Inhibits ITCH/AIP4-mediated ubiquitination of matrix protein 1 (M1) of IAV by impairing the interaction of ITCH/AIP4 with M1, followed by the suppression of the nuclear export of M1, and finally reduction of the replication of IAV (PubMed:30328013, PubMed:22347431). | activation of MAPK activity, activation of protein kinase B activity, apoptotic process, cell adhesion molecule production, cellular response to oxidative stress, endothelial cell activation, establishment of integrated proviral latency, fusion of virus membrane with host plasma membrane, interleukin-12-mediated signaling pathway, leukocyte chemotaxis, leukocyte migration, lipid droplet organization, negative regulation of oxidative stress-induced intrinsic apoptotic signaling pathway, negative regulation of protein K48-linked ubiquitination, negative regulation of protein kinase activity, negative regulation of protein phosphorylation, negative regulation of stress-activated MAPK cascade, negative regulation of viral life cycle, neutrophil chemotaxis, neutrophil degranulation, platelet activation, platelet aggregation, positive regulation of NF-kappaB transcription factor activity, positive regulation of protein dephosphorylation, positive regulation of protein phosphorylation, positive regulation of protein secretion, positive regulation of viral genome replication, protein folding, protein peptidyl-prolyl isomerization, regulation of apoptotic signaling pathway, regulation of viral genome replication, RNA-dependent DNA biosynthetic process, uncoating of virus, viral life cycle, viral release from host cell, virion assembly | rpsR | 30S ribosomal protein S18 | ribosome, rRNA binding, structural constituent of ribosome, translation |

**Table S4: Interactions between human and *E.coli* genes in polymicrobial infections and their corresponding protein functions.**

| **Human gene** | **Pathogen gene** | **Human full gene name** | **Human gene Function - from Uniprot** | **Function - from GO terms** | **Pathogen gene short name** | **Pathogen gene full name** | **Pathogen Function** |
| --- | --- | --- | --- | --- | --- | --- | --- |
| MATR3 | N2LMG4 | Matrin-3 | May play a role in transcription or may interact with other nuclear matrix proteins to form the internal fibrogranular network. In association with the SFPQ-NONO heteromer may play a role in nuclear retention of defective RNAs. Plays a role in the regulation of DNA virus-mediated innate immune response by assembling into the HDP-RNP complex, a complex that serves as a platform for IRF3 phosphorylation and subsequent innate immune response activation through the cGAS-STING pathway (PubMed:28712728). May bind to specific miRNA hairpins (PubMed:28431233). | activation of innate immune response, heart valve development, innate immune response, posttranscriptional regulation of gene expression, regulation of translation, ventricular septum development |  |  |  |
| ZNF302 | E9WNY3 | Zinc finger protein 302 | May be involved in transcriptional regulation. | regulation of transcription by RNA polymerase II |  |  |  |
| ZNF302 | F8XKK7 | Zinc finger protein 302 | May be involved in transcriptional regulation. | regulation of transcription by RNA polymerase II |  |  |  |
| ZNF302 | M4JP18 | Zinc finger protein 302 | May be involved in transcriptional regulation. | regulation of transcription by RNA polymerase II |  |  |  |
| ZNF302 | M7UZM1 | Zinc finger protein 302 | May be involved in transcriptional regulation. | regulation of transcription by RNA polymerase II |  |  |  |
| SORBS1 | T8QB09 | Sorbin and SH3 domain-containing protein 1 | Plays a role in tyrosine phosphorylation of CBL by linking CBL to the insulin receptor. Required for insulin-stimulated glucose transport. Involved in formation of actin stress fibers and focal adhesions (By similarity). | cell-matrix adhesion, cellular response to insulin stimulus, focal adhesion assembly, insulin receptor signaling pathway, muscle contraction, positive regulation of glucose import, positive regulation of glycogen biosynthetic process, positive regulation of insulin receptor signaling pathway, positive regulation of lipid biosynthetic process, positive regulation of protein localization to plasma membrane, stress fiber assembly |  |  |  |
| STAG2 | M4JP18 | Cohesin subunit SA-2 | Component of cohesin complex, a complex required for the cohesion of sister chromatids after DNA replication. The cohesin complex apparently forms a large proteinaceous ring within which sister chromatids can be trapped. At anaphase, the complex is cleaved and dissociates from chromatin, allowing sister chromatids to segregate. The cohesin complex may also play a role in spindle pole assembly during mitosis. | cell division, meiotic cell cycle, mitotic spindle assembly, sister chromatid cohesion |  |  |  |
| STAG2 | M7UZM1 | Cohesin subunit SA-2 | Component of cohesin complex, a complex required for the cohesion of sister chromatids after DNA replication. The cohesin complex apparently forms a large proteinaceous ring within which sister chromatids can be trapped. At anaphase, the complex is cleaved and dissociates from chromatin, allowing sister chromatids to segregate. The cohesin complex may also play a role in spindle pole assembly during mitosis. | cell division, meiotic cell cycle, mitotic spindle assembly, sister chromatid cohesion |  |  |  |
| BLOC1S6 | B3HKX7 | Biogenesis of lysosome-related organelles complex 1 subunit 6 | Component of the BLOC-1 complex, a complex that is required for normal biogenesis of lysosome-related organelles (LRO), such as platelet dense granules and melanosomes. In concert with the AP-3 complex, the BLOC-1 complex is required to target membrane protein cargos into vesicles assembled at cell bodies for delivery into neurites and nerve terminals. The BLOC-1 complex, in association with SNARE proteins, is also proposed to be involved in neurite extension. May play a role in intracellular vesicle trafficking, particularly in the vesicle-docking and fusion process. | anterograde axonal transport, anterograde synaptic vesicle transport, endosome to melanosome transport, intracellular transport, melanosome organization, melanosome transport, neuron projection development, positive regulation of pigment cell differentiation, synaptic vesicle docking |  |  |  |
| UBR5 | C1HUM8 | E3 ubiquitin-protein ligase UBR5 | E3 ubiquitin-protein ligase which is a component of the N-end rule pathway. Recognizes and binds to proteins bearing specific N-terminal residues that are destabilizing according to the N-end rule, leading to their ubiquitination and subsequent degradation (By similarity). Involved in maturation and/or transcriptional regulation of mRNA by activating CDK9 by polyubiquitination. May play a role in control of cell cycle progression. May have tumor suppressor function. Regulates DNA topoisomerase II binding protein (TopBP1) in the DNA damage response. Plays an essential role in extraembryonic development. Ubiquitinates acetylated PCK1. Also acts as a regulator of DNA damage response by acting as a suppressor of RNF168, an E3 ubiquitin-protein ligase that promotes accumulation of 'Lys-63'-linked histone H2A and H2AX at DNA damage sites, thereby acting as a guard against excessive spreading of ubiquitinated chromatin at damaged chromosomes. | cellular response to DNA damage stimulus, DNA repair, negative regulation of double-strand break repair, negative regulation of histone H2A K63-linked ubiquitination, positive regulation of canonical Wnt signaling pathway, positive regulation of gene expression, positive regulation of protein import into nucleus, progesterone receptor signaling pathway, protein K48-linked ubiquitination, protein polyubiquitination, regulation of double-strand break repair, viral process |  |  |  |
| ZPR1 | L1FQI3 | Zinc finger protein ZPR1 | Acts as a signaling molecule that communicates proliferative growth signals from the cytoplasm to the nucleus. Plays a role for the localization and accumulation of the survival motor neuron protein SMN1 in sub-nuclear bodies, including gems and Cajal bodies. Induces neuron differentiation and stimulates axonal growth and formation of growth cone in spinal cord motor neurons. Plays a role in the splicing of cellular pre-mRNAs. May be involved in H(2)O(2)-induced neuronal cell death. | apoptotic process involved in development, axon development, Cajal body organization, cell population proliferation, cellular response to epidermal growth factor stimulus, DNA endoreduplication, inner cell mass cell proliferation, microtubule cytoskeleton organization, mRNA processing, negative regulation of motor neuron apoptotic process, positive regulation of gene expression, positive regulation of growth, positive regulation of protein import into nucleus, positive regulation of RNA splicing, positive regulation of transcription involved in G1/S transition of mitotic cell cycle, pre-mRNA catabolic process, regulation of myelination, RNA splicing, signal transduction, spinal cord development, trophectodermal cell proliferation |  |  |  |
| EIF4G2 | F8XKK7 | Eukaryotic translation initiation factor 4 gamma 2 | Appears to play a role in the switch from cap-dependent to IRES-mediated translation during mitosis, apoptosis and viral infection. Cleaved by some caspases and viral proteases. | cell cycle arrest, cell death, cellular macromolecule biosynthetic process, heart development, negative regulation of autophagy, positive regulation of axon extension, positive regulation of cell growth, positive regulation of dendritic spine development, positive regulation of translation, regulation of translational initiation |  |  |  |
| EIF4G2 | S1L310 | Eukaryotic translation initiation factor 4 gamma 2 | Appears to play a role in the switch from cap-dependent to IRES-mediated translation during mitosis, apoptosis and viral infection. Cleaved by some caspases and viral proteases. | cell cycle arrest, cell death, cellular macromolecule biosynthetic process, heart development, negative regulation of autophagy, positive regulation of axon extension, positive regulation of cell growth, positive regulation of dendritic spine development, positive regulation of translation, regulation of translational initiation |  |  |  |
| FRK | B3HKX7 | Tyrosine-protein kinase FRK | Non-receptor tyrosine-protein kinase that negatively regulates cell proliferation. Positively regulates PTEN protein stability through phosphorylation of PTEN on 'Tyr-336', which in turn prevents its ubiquitination and degradation, possibly by reducing its binding to NEDD4. May function as a tumor suppressor. | cell differentiation, innate immune response, negative regulation of cell population proliferation, negative regulation of transcription by RNA polymerase II, neutrophil degranulation, peptidyl-tyrosine autophosphorylation, protein phosphorylation, regulation of cell population proliferation, transmembrane receptor protein tyrosine kinase signaling pathway |  |  |  |
| HINT3 | F8XKK7 | Histidine triad nucleotide-binding protein 3 | Hydrolyzes phosphoramidate and acyl-adenylate substrates. |  |  |  |  |
| HINT3 | S1L310 | Histidine triad nucleotide-binding protein 3 | Hydrolyzes phosphoramidate and acyl-adenylate substrates. |  |  |  |  |
| HINT3 | V2RYL8 | Histidine triad nucleotide-binding protein 3 | Hydrolyzes phosphoramidate and acyl-adenylate substrates. |  |  |  |  |
| GNAI2 | X2PD96 | Guanine nucleotide-binding protein G(i) subunit alpha-2 | Guanine nucleotide-binding proteins (G proteins) are involved as modulators or transducers in various transmembrane signaling systems. The G(i) proteins are involved in hormonal regulation of adenylate cyclase: they inhibit the cyclase in response to beta-adrenergic stimuli. May play a role in cell division. | adenylate cyclase-activating G protein-coupled receptor signaling pathway, adenylate cyclase-inhibiting G protein-coupled receptor signaling pathway, adenylate cyclase-modulating G protein-coupled receptor signaling pathway, cell cycle, cell division, cell population proliferation, G protein-coupled acetylcholine receptor signaling pathway, G protein-coupled adenosine receptor signaling pathway, G protein-coupled receptor signaling pathway, gamma-aminobutyric acid signaling pathway, negative regulation of adenylate cyclase activity, negative regulation of adenylate cyclase-activating adrenergic receptor signaling pathway involved in heart process, negative regulation of apoptotic signaling pathway, negative regulation of calcium ion-dependent exocytosis, negative regulation of protein tyrosine phosphatase activity, negative regulation of synaptic transmission, positive regulation of cell migration, positive regulation of cell population proliferation, positive regulation of ERK1 and ERK2 cascade, positive regulation of insulin receptor signaling pathway, positive regulation of NAD(P)H oxidase activity, positive regulation of neural precursor cell proliferation, positive regulation of renal sodium excretion, positive regulation of superoxide anion generation, positive regulation of urine volume, positive regulation of vascular associated smooth muscle cell proliferation, protein folding, regulation of calcium ion transport, response to nutrient, signal transduction |  |  |  |
| SF3B1 | N2LMG4 | Splicing factor 3B subunit 1 | Involved in pre-mRNA splicing as a component of the splicing factor SF3B complex (PubMed:27720643). SF3B complex is required for 'A' complex assembly formed by the stable binding of U2 snRNP to the branchpoint sequence (BPS) in pre-mRNA. Sequence independent binding of SF3A/SF3B complex upstream of the branch site is essential, it may anchor U2 snRNP to the pre-mRNA (PubMed:12234937). Together with other U2 snRNP complex components may also play a role in the selective processing of microRNAs (miRNAs) from the long primary miRNA transcript, pri-miR-17-92 (By similarity). May also be involved in the assembly of the 'E' complex (PubMed:10882114). Belongs also to the minor U12-dependent spliceosome, which is involved in the splicing of rare class of nuclear pre-mRNA intron (PubMed:15146077). | mRNA splicing, via spliceosome, positive regulation of gene expression, epigenetic, RNA splicing, RNA splicing, via transesterification reactions, spliceosomal complex assembly |  |  |  |
| RPAP2 | B3HKX7 | Putative RNA polymerase II subunit B1 CTD phosphatase RPAP2 | Protein phosphatase that displays CTD phosphatase activity and regulates transcription of snRNA genes. Recognizes and binds phosphorylated 'Ser-7' of the C-terminal heptapeptide repeat domain (CTD) of the largest RNA polymerase II subunit POLR2A, and mediates dephosphorylation of 'Ser-5' of the CTD, thereby promoting transcription of snRNA genes. | dephosphorylation of RNA polymerase II C-terminal domain, snRNA transcription, snRNA transcription by RNA polymerase II |  |  |  |
| MORC4 | B3HKX7 | MORC family CW-type zinc finger protein 4 | Histone methylation reader which binds to non-methylated (H3K4me0), monomethylated (H3K4me1), dimethylated (H3K4me2) and trimethylated (H3K4me3) 'Lys-4' on histone H3 (PubMed:26933034). The order of binding preference is H3K4me3 > H3K4me2 > H3K4me1 > H3K4me0 (PubMed:26933034). |  |  |  |  |
| TBC1D8B | B3HKX7 | TBC1 domain family member 8B | Involved in vesicular recycling, probably as a RAB11B GTPase-activating protein. | activation of GTPase activity, glomerular filtration, intracellular protein transport, vesicle-mediated transport |  |  |  |
| FAM83A | E1IVY0 | Protein FAM83A | Probable proto-oncogene that functions in the epidermal growth factor receptor/EGFR signaling pathway. Activates both RAS/MAPK and PI3K/AKT/TOR signaling cascades downstream of EGFR. Required for the RAS/MAPK signaling cascade activation upon EGFR stimulation, it also activates both signaling cascades independently of EGFR activation. | cell population proliferation, epidermal growth factor receptor signaling pathway, signal transduction | HMPREF9348_04367 | Uncharacterized protein |  |
| FAM83A | E9WNY3 | Protein FAM83A | Probable proto-oncogene that functions in the epidermal growth factor receptor/EGFR signaling pathway. Activates both RAS/MAPK and PI3K/AKT/TOR signaling cascades downstream of EGFR. Required for the RAS/MAPK signaling cascade activation upon EGFR stimulation, it also activates both signaling cascades independently of EGFR activation. | cell population proliferation, epidermal growth factor receptor signaling pathway, signal transduction |  |  |  |
| FAM83A | F8XKK7 | Protein FAM83A | Probable proto-oncogene that functions in the epidermal growth factor receptor/EGFR signaling pathway. Activates both RAS/MAPK and PI3K/AKT/TOR signaling cascades downstream of EGFR. Required for the RAS/MAPK signaling cascade activation upon EGFR stimulation, it also activates both signaling cascades independently of EGFR activation. | cell population proliferation, epidermal growth factor receptor signaling pathway, signal transduction |  |  |  |
| FAM83A | M4JP18 | Protein FAM83A | Probable proto-oncogene that functions in the epidermal growth factor receptor/EGFR signaling pathway. Activates both RAS/MAPK and PI3K/AKT/TOR signaling cascades downstream of EGFR. Required for the RAS/MAPK signaling cascade activation upon EGFR stimulation, it also activates both signaling cascades independently of EGFR activation. | cell population proliferation, epidermal growth factor receptor signaling pathway, signal transduction |  |  |  |
| FAM83A | M7UZM1 | Protein FAM83A | Probable proto-oncogene that functions in the epidermal growth factor receptor/EGFR signaling pathway. Activates both RAS/MAPK and PI3K/AKT/TOR signaling cascades downstream of EGFR. Required for the RAS/MAPK signaling cascade activation upon EGFR stimulation, it also activates both signaling cascades independently of EGFR activation. | cell population proliferation, epidermal growth factor receptor signaling pathway, signal transduction |  |  |  |
| FAM83A | S1L310 | Protein FAM83A | Probable proto-oncogene that functions in the epidermal growth factor receptor/EGFR signaling pathway. Activates both RAS/MAPK and PI3K/AKT/TOR signaling cascades downstream of EGFR. Required for the RAS/MAPK signaling cascade activation upon EGFR stimulation, it also activates both signaling cascades independently of EGFR activation. | cell population proliferation, epidermal growth factor receptor signaling pathway, signal transduction |  |  |  |
| FAM83A | X3F781 | Protein FAM83A | Probable proto-oncogene that functions in the epidermal growth factor receptor/EGFR signaling pathway. Activates both RAS/MAPK and PI3K/AKT/TOR signaling cascades downstream of EGFR. Required for the RAS/MAPK signaling cascade activation upon EGFR stimulation, it also activates both signaling cascades independently of EGFR activation. | cell population proliferation, epidermal growth factor receptor signaling pathway, signal transduction |  |  |  |
| ASTN2 | B3HKX7 | Astrotactin-2 | Mediates recycling of the neuronal cell adhesion molecule ASTN1 to the anterior pole of the cell membrane in migrating neurons. Promotes ASTN1 internalization and intracellular transport of endocytosed ASTN1 (By similarity). Selectively binds inositol-4,5-bisphosphate, inositol-3,4,5-trisphosphate and inositol-1,3,4,5-tetrakisphosphate, suggesting it is recruited to membranes that contain lipids with a phosphoinositide headgroup (Ref.6). | establishment of body hair planar orientation, negative regulation of protein localization to cell surface, neuron cell-cell adhesion, neuron migration, protein transport |  |  |  |
| RPL7A | E1IVY0 | 60S ribosomal protein L7a |  | maturation of LSU-rRNA, nuclear-transcribed mRNA catabolic process, nonsense-mediated decay, SRP-dependent cotranslational protein targeting to membrane, translation, translational initiation, viral transcription | HMPREF9348_04367 | Uncharacterized protein |  |
| RPL7A | E9WNY3 | 60S ribosomal protein L7a |  | maturation of LSU-rRNA, nuclear-transcribed mRNA catabolic process, nonsense-mediated decay, SRP-dependent cotranslational protein targeting to membrane, translation, translational initiation, viral transcription |  |  |  |
| RPL7A | F8XKK7 | 60S ribosomal protein L7a |  | maturation of LSU-rRNA, nuclear-transcribed mRNA catabolic process, nonsense-mediated decay, SRP-dependent cotranslational protein targeting to membrane, translation, translational initiation, viral transcription |  |  |  |
| RPL7A | H4S5L1 | 60S ribosomal protein L7a |  | maturation of LSU-rRNA, nuclear-transcribed mRNA catabolic process, nonsense-mediated decay, SRP-dependent cotranslational protein targeting to membrane, translation, translational initiation, viral transcription |  |  |  |
| RPL7A | H5G4G2 | 60S ribosomal protein L7a |  | maturation of LSU-rRNA, nuclear-transcribed mRNA catabolic process, nonsense-mediated decay, SRP-dependent cotranslational protein targeting to membrane, translation, translational initiation, viral transcription |  |  |  |
| RPL7A | M4JP18 | 60S ribosomal protein L7a |  | maturation of LSU-rRNA, nuclear-transcribed mRNA catabolic process, nonsense-mediated decay, SRP-dependent cotranslational protein targeting to membrane, translation, translational initiation, viral transcription |  |  |  |
| RPL7A | M7UZM1 | 60S ribosomal protein L7a |  | maturation of LSU-rRNA, nuclear-transcribed mRNA catabolic process, nonsense-mediated decay, SRP-dependent cotranslational protein targeting to membrane, translation, translational initiation, viral transcription |  |  |  |
| RPL7A | S1L310 | 60S ribosomal protein L7a |  | maturation of LSU-rRNA, nuclear-transcribed mRNA catabolic process, nonsense-mediated decay, SRP-dependent cotranslational protein targeting to membrane, translation, translational initiation, viral transcription |  |  |  |
| RPL7A | T6RN53 | 60S ribosomal protein L7a |  | maturation of LSU-rRNA, nuclear-transcribed mRNA catabolic process, nonsense-mediated decay, SRP-dependent cotranslational protein targeting to membrane, translation, translational initiation, viral transcription |  |  |  |
| RPL7A | X3F781 | 60S ribosomal protein L7a |  | maturation of LSU-rRNA, nuclear-transcribed mRNA catabolic process, nonsense-mediated decay, SRP-dependent cotranslational protein targeting to membrane, translation, translational initiation, viral transcription |  |  |  |
| KIAA1328 | B3HKX7 | Protein hinderin | Competes with SMC1 for binding to SMC3. May affect the availability of SMC3 to engage in the formation of multimeric protein complexes. |  |  |  |  |
| C12orf45 | B3HKX7 | Uncharacterized protein C12orf45 |  |  |  |  |  |
| MIPOL1 | B3HKX7 | Mirror-image polydactyly gene 1 protein |  |  |  |  |  |
| LRRK1 | B3HKX7 | Leucine-rich repeat serine/threonine-protein kinase 1 |  | bone resorption, negative regulation of peptidyl-tyrosine phosphorylation, osteoclast development, positive regulation of canonical Wnt signaling pathway, positive regulation of intracellular signal transduction, positive regulation of peptidyl-tyrosine phosphorylation, signal transduction |  |  |  |
| EIF4A2 | C1HUN3 | Eukaryotic initiation factor 4A-II | ATP-dependent RNA helicase which is a subunit of the eIF4F complex involved in cap recognition and is required for mRNA binding to ribosome. In the current model of translation initiation, eIF4A unwinds RNA secondary structures in the 5'-UTR of mRNAs which is necessary to allow efficient binding of the small ribosomal subunit, and subsequent scanning for the initiator codon. | cellular response to leukemia inhibitory factor, cytoplasmic translational initiation, negative regulation of RNA-directed 5'-3' RNA polymerase activity, regulation of translational initiation, translational initiation, viral process |  |  |  |
| EIF4A2 | E1IVY0 | Eukaryotic initiation factor 4A-II | ATP-dependent RNA helicase which is a subunit of the eIF4F complex involved in cap recognition and is required for mRNA binding to ribosome. In the current model of translation initiation, eIF4A unwinds RNA secondary structures in the 5'-UTR of mRNAs which is necessary to allow efficient binding of the small ribosomal subunit, and subsequent scanning for the initiator codon. | cellular response to leukemia inhibitory factor, cytoplasmic translational initiation, negative regulation of RNA-directed 5'-3' RNA polymerase activity, regulation of translational initiation, translational initiation, viral process | HMPREF9348_04367 | Uncharacterized protein |  |
| EIF4A2 | E9WNY3 | Eukaryotic initiation factor 4A-II | ATP-dependent RNA helicase which is a subunit of the eIF4F complex involved in cap recognition and is required for mRNA binding to ribosome. In the current model of translation initiation, eIF4A unwinds RNA secondary structures in the 5'-UTR of mRNAs which is necessary to allow efficient binding of the small ribosomal subunit, and subsequent scanning for the initiator codon. | cellular response to leukemia inhibitory factor, cytoplasmic translational initiation, negative regulation of RNA-directed 5'-3' RNA polymerase activity, regulation of translational initiation, translational initiation, viral process |  |  |  |
| EIF4A2 | F8XKK7 | Eukaryotic initiation factor 4A-II | ATP-dependent RNA helicase which is a subunit of the eIF4F complex involved in cap recognition and is required for mRNA binding to ribosome. In the current model of translation initiation, eIF4A unwinds RNA secondary structures in the 5'-UTR of mRNAs which is necessary to allow efficient binding of the small ribosomal subunit, and subsequent scanning for the initiator codon. | cellular response to leukemia inhibitory factor, cytoplasmic translational initiation, negative regulation of RNA-directed 5'-3' RNA polymerase activity, regulation of translational initiation, translational initiation, viral process |  |  |  |
| EIF4A2 | H4S5N3 | Eukaryotic initiation factor 4A-II | ATP-dependent RNA helicase which is a subunit of the eIF4F complex involved in cap recognition and is required for mRNA binding to ribosome. In the current model of translation initiation, eIF4A unwinds RNA secondary structures in the 5'-UTR of mRNAs which is necessary to allow efficient binding of the small ribosomal subunit, and subsequent scanning for the initiator codon. | cellular response to leukemia inhibitory factor, cytoplasmic translational initiation, negative regulation of RNA-directed 5'-3' RNA polymerase activity, regulation of translational initiation, translational initiation, viral process |  |  |  |
| EIF4A2 | H5G4G2 | Eukaryotic initiation factor 4A-II | ATP-dependent RNA helicase which is a subunit of the eIF4F complex involved in cap recognition and is required for mRNA binding to ribosome. In the current model of translation initiation, eIF4A unwinds RNA secondary structures in the 5'-UTR of mRNAs which is necessary to allow efficient binding of the small ribosomal subunit, and subsequent scanning for the initiator codon. | cellular response to leukemia inhibitory factor, cytoplasmic translational initiation, negative regulation of RNA-directed 5'-3' RNA polymerase activity, regulation of translational initiation, translational initiation, viral process |  |  |  |
| EIF4A2 | J7QIY4 | Eukaryotic initiation factor 4A-II | ATP-dependent RNA helicase which is a subunit of the eIF4F complex involved in cap recognition and is required for mRNA binding to ribosome. In the current model of translation initiation, eIF4A unwinds RNA secondary structures in the 5'-UTR of mRNAs which is necessary to allow efficient binding of the small ribosomal subunit, and subsequent scanning for the initiator codon. | cellular response to leukemia inhibitory factor, cytoplasmic translational initiation, negative regulation of RNA-directed 5'-3' RNA polymerase activity, regulation of translational initiation, translational initiation, viral process | BN17_38010 | Uncharacterized protein |  |
| EIF4A2 | L1FQI3 | Eukaryotic initiation factor 4A-II | ATP-dependent RNA helicase which is a subunit of the eIF4F complex involved in cap recognition and is required for mRNA binding to ribosome. In the current model of translation initiation, eIF4A unwinds RNA secondary structures in the 5'-UTR of mRNAs which is necessary to allow efficient binding of the small ribosomal subunit, and subsequent scanning for the initiator codon. | cellular response to leukemia inhibitory factor, cytoplasmic translational initiation, negative regulation of RNA-directed 5'-3' RNA polymerase activity, regulation of translational initiation, translational initiation, viral process |  |  |  |
| EIF4A2 | L2CS94 | Eukaryotic initiation factor 4A-II | ATP-dependent RNA helicase which is a subunit of the eIF4F complex involved in cap recognition and is required for mRNA binding to ribosome. In the current model of translation initiation, eIF4A unwinds RNA secondary structures in the 5'-UTR of mRNAs which is necessary to allow efficient binding of the small ribosomal subunit, and subsequent scanning for the initiator codon. | cellular response to leukemia inhibitory factor, cytoplasmic translational initiation, negative regulation of RNA-directed 5'-3' RNA polymerase activity, regulation of translational initiation, translational initiation, viral process |  |  |  |
| EIF4A2 | M4JP18 | Eukaryotic initiation factor 4A-II | ATP-dependent RNA helicase which is a subunit of the eIF4F complex involved in cap recognition and is required for mRNA binding to ribosome. In the current model of translation initiation, eIF4A unwinds RNA secondary structures in the 5'-UTR of mRNAs which is necessary to allow efficient binding of the small ribosomal subunit, and subsequent scanning for the initiator codon. | cellular response to leukemia inhibitory factor, cytoplasmic translational initiation, negative regulation of RNA-directed 5'-3' RNA polymerase activity, regulation of translational initiation, translational initiation, viral process |  |  |  |
| EIF4A2 | R9VPP3 | Eukaryotic initiation factor 4A-II | ATP-dependent RNA helicase which is a subunit of the eIF4F complex involved in cap recognition and is required for mRNA binding to ribosome. In the current model of translation initiation, eIF4A unwinds RNA secondary structures in the 5'-UTR of mRNAs which is necessary to allow efficient binding of the small ribosomal subunit, and subsequent scanning for the initiator codon. | cellular response to leukemia inhibitory factor, cytoplasmic translational initiation, negative regulation of RNA-directed 5'-3' RNA polymerase activity, regulation of translational initiation, translational initiation, viral process | H650_16415 | Uncharacterized protein |  |
| EIF4A2 | S1L310 | Eukaryotic initiation factor 4A-II | ATP-dependent RNA helicase which is a subunit of the eIF4F complex involved in cap recognition and is required for mRNA binding to ribosome. In the current model of translation initiation, eIF4A unwinds RNA secondary structures in the 5'-UTR of mRNAs which is necessary to allow efficient binding of the small ribosomal subunit, and subsequent scanning for the initiator codon. | cellular response to leukemia inhibitory factor, cytoplasmic translational initiation, negative regulation of RNA-directed 5'-3' RNA polymerase activity, regulation of translational initiation, translational initiation, viral process |  |  |  |
| EIF4A2 | T6RN53 | Eukaryotic initiation factor 4A-II | ATP-dependent RNA helicase which is a subunit of the eIF4F complex involved in cap recognition and is required for mRNA binding to ribosome. In the current model of translation initiation, eIF4A unwinds RNA secondary structures in the 5'-UTR of mRNAs which is necessary to allow efficient binding of the small ribosomal subunit, and subsequent scanning for the initiator codon. | cellular response to leukemia inhibitory factor, cytoplasmic translational initiation, negative regulation of RNA-directed 5'-3' RNA polymerase activity, regulation of translational initiation, translational initiation, viral process |  |  |  |
| EIF4A2 | X2PD96 | Eukaryotic initiation factor 4A-II | ATP-dependent RNA helicase which is a subunit of the eIF4F complex involved in cap recognition and is required for mRNA binding to ribosome. In the current model of translation initiation, eIF4A unwinds RNA secondary structures in the 5'-UTR of mRNAs which is necessary to allow efficient binding of the small ribosomal subunit, and subsequent scanning for the initiator codon. | cellular response to leukemia inhibitory factor, cytoplasmic translational initiation, negative regulation of RNA-directed 5'-3' RNA polymerase activity, regulation of translational initiation, translational initiation, viral process |  |  |  |
| TAF1D | E9WNY3 | TATA box-binding protein-associated factor RNA polymerase I subunit D | Component of the transcription factor SL1/TIF-IB complex, which is involved in the assembly of the PIC (preinitiation complex) during RNA polymerase I-dependent transcription. The rate of PIC formation probably is primarily dependent on the rate of association of SL1/TIF-IB with the rDNA promoter. SL1/TIF-IB is involved in stabilization of nucleolar transcription factor 1/UBTF on rDNA. Formation of SL1/TIF-IB excludes the association of TBP with TFIID subunits. | positive regulation of gene expression, epigenetic, regulation of transcription, DNA-templated, termination of RNA polymerase I transcription, transcription elongation from RNA polymerase I promoter, transcription initiation from RNA polymerase I promoter |  |  |  |
| TAF1D | L1FQI3 | TATA box-binding protein-associated factor RNA polymerase I subunit D | Component of the transcription factor SL1/TIF-IB complex, which is involved in the assembly of the PIC (preinitiation complex) during RNA polymerase I-dependent transcription. The rate of PIC formation probably is primarily dependent on the rate of association of SL1/TIF-IB with the rDNA promoter. SL1/TIF-IB is involved in stabilization of nucleolar transcription factor 1/UBTF on rDNA. Formation of SL1/TIF-IB excludes the association of TBP with TFIID subunits. | positive regulation of gene expression, epigenetic, regulation of transcription, DNA-templated, termination of RNA polymerase I transcription, transcription elongation from RNA polymerase I promoter, transcription initiation from RNA polymerase I promoter |  |  |  |
| TAF1D | M4JP18 | TATA box-binding protein-associated factor RNA polymerase I subunit D | Component of the transcription factor SL1/TIF-IB complex, which is involved in the assembly of the PIC (preinitiation complex) during RNA polymerase I-dependent transcription. The rate of PIC formation probably is primarily dependent on the rate of association of SL1/TIF-IB with the rDNA promoter. SL1/TIF-IB is involved in stabilization of nucleolar transcription factor 1/UBTF on rDNA. Formation of SL1/TIF-IB excludes the association of TBP with TFIID subunits. | positive regulation of gene expression, epigenetic, regulation of transcription, DNA-templated, termination of RNA polymerase I transcription, transcription elongation from RNA polymerase I promoter, transcription initiation from RNA polymerase I promoter |  |  |  |
| TAF1D | M7UZM1 | TATA box-binding protein-associated factor RNA polymerase I subunit D | Component of the transcription factor SL1/TIF-IB complex, which is involved in the assembly of the PIC (preinitiation complex) during RNA polymerase I-dependent transcription. The rate of PIC formation probably is primarily dependent on the rate of association of SL1/TIF-IB with the rDNA promoter. SL1/TIF-IB is involved in stabilization of nucleolar transcription factor 1/UBTF on rDNA. Formation of SL1/TIF-IB excludes the association of TBP with TFIID subunits. | positive regulation of gene expression, epigenetic, regulation of transcription, DNA-templated, termination of RNA polymerase I transcription, transcription elongation from RNA polymerase I promoter, transcription initiation from RNA polymerase I promoter |  |  |  |
| TAF1D | S1L310 | TATA box-binding protein-associated factor RNA polymerase I subunit D | Component of the transcription factor SL1/TIF-IB complex, which is involved in the assembly of the PIC (preinitiation complex) during RNA polymerase I-dependent transcription. The rate of PIC formation probably is primarily dependent on the rate of association of SL1/TIF-IB with the rDNA promoter. SL1/TIF-IB is involved in stabilization of nucleolar transcription factor 1/UBTF on rDNA. Formation of SL1/TIF-IB excludes the association of TBP with TFIID subunits. | positive regulation of gene expression, epigenetic, regulation of transcription, DNA-templated, termination of RNA polymerase I transcription, transcription elongation from RNA polymerase I promoter, transcription initiation from RNA polymerase I promoter |  |  |  |
| ATP9B | B3HKX7 | Probable phospholipid-transporting ATPase IIB |  | endocytosis, phospholipid translocation, retrograde vesicle-mediated transport, Golgi to endoplasmic reticulum |  |  |  |
| SRRM2 | N2LMG4 | Serine/arginine repetitive matrix protein 2 | Required for pre-mRNA splicing as component of the spliceosome. | mRNA splicing, via spliceosome |  |  |  |
| CYSLTR1 | B3HKX7 | Cysteinyl leukotriene receptor 1 | Receptor for cysteinyl leukotrienes mediating bronchoconstriction of individuals with and without asthma. Stimulation by LTD4 results in the contraction and proliferation of smooth muscle, edema, eosinophil migration and damage to the mucus layer in the lung. This response is mediated via a G-protein that activates a phosphatidylinositol-calcium second messenger system. The rank order of affinities for the leukotrienes is LTD4 >> LTE4 = LTC4 >> LTB4. | calcium ion transport, cell surface receptor signaling pathway, chemotaxis, defense response, G protein-coupled receptor signaling pathway, inflammatory response to antigenic stimulus, neuropeptide signaling pathway, positive regulation of cytosolic calcium ion concentration, respiratory gaseous exchange by respiratory system |  |  |  |
| SULT1B1 | B3HKX7 | Sulfotransferase family cytosolic 1B member 1 | Sulfotransferase that utilizes 3'-phospho-5'-adenylyl sulfate (PAPS) as sulfonate donor to catalyze the sulfate conjugation of many hormones, neurotransmitters, drugs and xenobiotic compounds. Sulfonation increases the water solubility of most compounds, and therefore their renal excretion, but it can also result in bioactivation to form active metabolites. Sulfates dopamine, small phenols such as 1-naphthol and p-nitrophenol and thyroid hormones, including 3,3'-diiodothyronine, triidothyronine, reverse triiodothyronine and thyroxine. | 3'-phosphoadenosine 5'-phosphosulfate metabolic process, cellular biogenic amine metabolic process, epithelial cell differentiation, ethanol catabolic process, flavonoid metabolic process, phenol-containing compound metabolic process, steroid metabolic process, sulfation, thyroid hormone metabolic process, xenobiotic metabolic process |  |  |  |
| FAM241A | B3HKX7 | Uncharacterized protein FAM241A |  |  |  |  |  |
| TMEM45A | B3HKX7 | Transmembrane protein 45A |  |  |  |  |  |
| ANXA2 | E1IVY0 | Annexin A2 | Calcium-regulated membrane-binding protein whose affinity for calcium is greatly enhanced by anionic phospholipids. It binds two calcium ions with high affinity. May be involved in heat-stress response. Inhibits PCSK9-enhanced LDLR degradation, probably reduces PCSK9 protein levels via a translational mechanism but also competes with LDLR for binding with PCSK9 (PubMed:18799458, PubMed:24808179, PubMed:22848640). | angiogenesis, catabolism by host of symbiont protein, interleukin-12-mediated signaling pathway, membrane raft assembly, negative regulation by host of symbiont molecular function, negative regulation of development of symbiont involved in interaction with host, negative regulation of low-density lipoprotein particle receptor catabolic process, neutrophil degranulation, osteoclast development, positive regulation of low-density lipoprotein particle clearance, positive regulation of low-density lipoprotein particle receptor binding, positive regulation of low-density lipoprotein receptor activity, positive regulation of receptor recycling, positive regulation of receptor-mediated endocytosis involved in cholesterol transport, positive regulation of vacuole organization, positive regulation of vesicle fusion, vesicle budding from membrane, viral process | HMPREF9348_04367 | Uncharacterized protein |  |
| ANXA2 | H4S5N3 | Annexin A2 | Calcium-regulated membrane-binding protein whose affinity for calcium is greatly enhanced by anionic phospholipids. It binds two calcium ions with high affinity. May be involved in heat-stress response. Inhibits PCSK9-enhanced LDLR degradation, probably reduces PCSK9 protein levels via a translational mechanism but also competes with LDLR for binding with PCSK9 (PubMed:18799458, PubMed:24808179, PubMed:22848640). | angiogenesis, catabolism by host of symbiont protein, interleukin-12-mediated signaling pathway, membrane raft assembly, negative regulation by host of symbiont molecular function, negative regulation of development of symbiont involved in interaction with host, negative regulation of low-density lipoprotein particle receptor catabolic process, neutrophil degranulation, osteoclast development, positive regulation of low-density lipoprotein particle clearance, positive regulation of low-density lipoprotein particle receptor binding, positive regulation of low-density lipoprotein receptor activity, positive regulation of receptor recycling, positive regulation of receptor-mediated endocytosis involved in cholesterol transport, positive regulation of vacuole organization, positive regulation of vesicle fusion, vesicle budding from membrane, viral process |  |  |  |
| ANXA2 | R9VPP3 | Annexin A2 | Calcium-regulated membrane-binding protein whose affinity for calcium is greatly enhanced by anionic phospholipids. It binds two calcium ions with high affinity. May be involved in heat-stress response. Inhibits PCSK9-enhanced LDLR degradation, probably reduces PCSK9 protein levels via a translational mechanism but also competes with LDLR for binding with PCSK9 (PubMed:18799458, PubMed:24808179, PubMed:22848640). | angiogenesis, catabolism by host of symbiont protein, interleukin-12-mediated signaling pathway, membrane raft assembly, negative regulation by host of symbiont molecular function, negative regulation of development of symbiont involved in interaction with host, negative regulation of low-density lipoprotein particle receptor catabolic process, neutrophil degranulation, osteoclast development, positive regulation of low-density lipoprotein particle clearance, positive regulation of low-density lipoprotein particle receptor binding, positive regulation of low-density lipoprotein receptor activity, positive regulation of receptor recycling, positive regulation of receptor-mediated endocytosis involved in cholesterol transport, positive regulation of vacuole organization, positive regulation of vesicle fusion, vesicle budding from membrane, viral process | H650_16415 | Uncharacterized protein |  |
| ACTG1 | I2RV19 | Actin, cytoplasmic 2 | Actins are highly conserved proteins that are involved in various types of cell motility and are ubiquitously expressed in all eukaryotic cells. | angiogenesis, cell junction assembly, cellular response to interferon-gamma, ephrin receptor signaling pathway, Fc-gamma receptor signaling pathway involved in phagocytosis, maintenance of blood-brain barrier, membrane organization, morphogenesis of a polarized epithelium, platelet aggregation, positive regulation of cell migration, positive regulation of gene expression, positive regulation of wound healing, protein localization to bicellular tight junction, regulation of focal adhesion assembly, regulation of stress fiber assembly, regulation of transepithelial transport, retina homeostasis, sarcomere organization, synaptic vesicle endocytosis, tight junction assembly | EC970246_D0002 | Uncharacterized protein |  |
| CFAP54 | B3HKX7 | Cilia- and flagella-associated protein 54 | Required for assembly and function of cilia and flagella. | cell differentiation, cilium assembly, cilium movement involved in cell motility, spermatogenesis |  |  |  |
| KPNA5 | B3HKX7 | Importin subunit alpha-6 | Functions in nuclear protein import as an adapter protein for nuclear receptor KPNB1. Binds specifically and directly to substrates containing either a simple or bipartite NLS motif. Docking of the importin/substrate complex to the nuclear pore complex (NPC) is mediated by KPNB1 through binding to nucleoporin FxFG repeats and the complex is subsequently translocated through the pore by an energy requiring, Ran-dependent mechanism. At the nucleoplasmic side of the NPC, Ran binds to importin-beta and the three components separate and importin-alpha and -beta are re-exported from the nucleus to the cytoplasm where GTP hydrolysis releases Ran from importin. The directionality of nuclear import is thought to be conferred by an asymmetric distribution of the GTP- and GDP-bound forms of Ran between the cytoplasm and nucleus. Mediates nuclear import of STAT1 homodimers and STAT1/STAT2 heterodimers by recognizing non-classical NLSs of STAT1 and STAT2 through ARM repeats 8-9. Recognizes influenza A virus nucleoprotein through ARM repeat 7-9 In vitro, mediates the nuclear import of human cytomegalovirus UL84 by recognizing a non-classical NLS. | modulation by virus of host cellular process, NLS-bearing protein import into nucleus |  |  |  |
| MTRNR2L8 | A0A023KWQ1 | Humanin-like 8 | Plays a role as a neuroprotective and antiapoptotic factor. | negative regulation of execution phase of apoptosis |  |  |  |
| MTRNR2L8 | C1HUM8 | Humanin-like 8 | Plays a role as a neuroprotective and antiapoptotic factor. | negative regulation of execution phase of apoptosis |  |  |  |
| MTRNR2L8 | C1HUN3 | Humanin-like 8 | Plays a role as a neuroprotective and antiapoptotic factor. | negative regulation of execution phase of apoptosis |  |  |  |
| MTRNR2L8 | E1IVY0 | Humanin-like 8 | Plays a role as a neuroprotective and antiapoptotic factor. | negative regulation of execution phase of apoptosis | HMPREF9348_04367 | Uncharacterized protein |  |
| MTRNR2L8 | E9WNY3 | Humanin-like 8 | Plays a role as a neuroprotective and antiapoptotic factor. | negative regulation of execution phase of apoptosis |  |  |  |
| MTRNR2L8 | F8XKK7 | Humanin-like 8 | Plays a role as a neuroprotective and antiapoptotic factor. | negative regulation of execution phase of apoptosis |  |  |  |
| MTRNR2L8 | H4M7I0 | Humanin-like 8 | Plays a role as a neuroprotective and antiapoptotic factor. | negative regulation of execution phase of apoptosis |  |  |  |
| MTRNR2L8 | H4S5L1 | Humanin-like 8 | Plays a role as a neuroprotective and antiapoptotic factor. | negative regulation of execution phase of apoptosis |  |  |  |
| MTRNR2L8 | H4S5N3 | Humanin-like 8 | Plays a role as a neuroprotective and antiapoptotic factor. | negative regulation of execution phase of apoptosis |  |  |  |
| MTRNR2L8 | H5G4G2 | Humanin-like 8 | Plays a role as a neuroprotective and antiapoptotic factor. | negative regulation of execution phase of apoptosis |  |  |  |
| MTRNR2L8 | J7QIY4 | Humanin-like 8 | Plays a role as a neuroprotective and antiapoptotic factor. | negative regulation of execution phase of apoptosis | BN17_38010 | Uncharacterized protein |  |
| MTRNR2L8 | L1FQI3 | Humanin-like 8 | Plays a role as a neuroprotective and antiapoptotic factor. | negative regulation of execution phase of apoptosis |  |  |  |
| MTRNR2L8 | L2CS94 | Humanin-like 8 | Plays a role as a neuroprotective and antiapoptotic factor. | negative regulation of execution phase of apoptosis |  |  |  |
| MTRNR2L8 | Q57T05 | Humanin-like 8 | Plays a role as a neuroprotective and antiapoptotic factor. | negative regulation of execution phase of apoptosis | SCH_0250 | Uncharacterized protein |  |
| MTRNR2L8 | R9VPP3 | Humanin-like 8 | Plays a role as a neuroprotective and antiapoptotic factor. | negative regulation of execution phase of apoptosis | H650_16415 | Uncharacterized protein |  |
| MTRNR2L8 | S1L310 | Humanin-like 8 | Plays a role as a neuroprotective and antiapoptotic factor. | negative regulation of execution phase of apoptosis |  |  |  |
| MTRNR2L8 | T6RN53 | Humanin-like 8 | Plays a role as a neuroprotective and antiapoptotic factor. | negative regulation of execution phase of apoptosis |  |  |  |
| MTRNR2L8 | T8B832 | Humanin-like 8 | Plays a role as a neuroprotective and antiapoptotic factor. | negative regulation of execution phase of apoptosis |  |  |  |
| MTRNR2L8 | U0HCA7 | Humanin-like 8 | Plays a role as a neuroprotective and antiapoptotic factor. | negative regulation of execution phase of apoptosis |  |  |  |
| MTRNR2L8 | V2RYL8 | Humanin-like 8 | Plays a role as a neuroprotective and antiapoptotic factor. | negative regulation of execution phase of apoptosis |  |  |  |
| MTRNR2L8 | X2PD96 | Humanin-like 8 | Plays a role as a neuroprotective and antiapoptotic factor. | negative regulation of execution phase of apoptosis |  |  |  |
| MTRNR2L8 | X3F781 | Humanin-like 8 | Plays a role as a neuroprotective and antiapoptotic factor. | negative regulation of execution phase of apoptosis |  |  |  |
| MTRNR2L1 | A0A023KWQ1 | Humanin-like 1 | Plays a role as a neuroprotective and antiapoptotic factor. | negative regulation of execution phase of apoptosis |  |  |  |
| MTRNR2L1 | E1IVY0 | Humanin-like 1 | Plays a role as a neuroprotective and antiapoptotic factor. | negative regulation of execution phase of apoptosis | HMPREF9348_04367 | Uncharacterized protein |  |
| MTRNR2L1 | E9WNY3 | Humanin-like 1 | Plays a role as a neuroprotective and antiapoptotic factor. | negative regulation of execution phase of apoptosis |  |  |  |
| MTRNR2L1 | F8XKK7 | Humanin-like 1 | Plays a role as a neuroprotective and antiapoptotic factor. | negative regulation of execution phase of apoptosis |  |  |  |
| MTRNR2L1 | H4M7I0 | Humanin-like 1 | Plays a role as a neuroprotective and antiapoptotic factor. | negative regulation of execution phase of apoptosis |  |  |  |
| MTRNR2L1 | H4S5L1 | Humanin-like 1 | Plays a role as a neuroprotective and antiapoptotic factor. | negative regulation of execution phase of apoptosis |  |  |  |
| MTRNR2L1 | H4S5N3 | Humanin-like 1 | Plays a role as a neuroprotective and antiapoptotic factor. | negative regulation of execution phase of apoptosis |  |  |  |
| MTRNR2L1 | H5G4G2 | Humanin-like 1 | Plays a role as a neuroprotective and antiapoptotic factor. | negative regulation of execution phase of apoptosis |  |  |  |
| MTRNR2L1 | J7QIY4 | Humanin-like 1 | Plays a role as a neuroprotective and antiapoptotic factor. | negative regulation of execution phase of apoptosis | BN17_38010 | Uncharacterized protein |  |
| MTRNR2L1 | L2CS94 | Humanin-like 1 | Plays a role as a neuroprotective and antiapoptotic factor. | negative regulation of execution phase of apoptosis |  |  |  |
| MTRNR2L1 | Q57T05 | Humanin-like 1 | Plays a role as a neuroprotective and antiapoptotic factor. | negative regulation of execution phase of apoptosis | SCH_0250 | Uncharacterized protein |  |
| MTRNR2L1 | R9VPP3 | Humanin-like 1 | Plays a role as a neuroprotective and antiapoptotic factor. | negative regulation of execution phase of apoptosis | H650_16415 | Uncharacterized protein |  |
| MTRNR2L1 | S1L310 | Humanin-like 1 | Plays a role as a neuroprotective and antiapoptotic factor. | negative regulation of execution phase of apoptosis |  |  |  |
| MTRNR2L1 | T6RN53 | Humanin-like 1 | Plays a role as a neuroprotective and antiapoptotic factor. | negative regulation of execution phase of apoptosis |  |  |  |
| MTRNR2L1 | U0HCA7 | Humanin-like 1 | Plays a role as a neuroprotective and antiapoptotic factor. | negative regulation of execution phase of apoptosis |  |  |  |
| MTRNR2L1 | V2RYL8 | Humanin-like 1 | Plays a role as a neuroprotective and antiapoptotic factor. | negative regulation of execution phase of apoptosis |  |  |  |
| MTRNR2L1 | V8TC87 | Humanin-like 1 | Plays a role as a neuroprotective and antiapoptotic factor. | negative regulation of execution phase of apoptosis |  |  |  |
| MTRNR2L1 | X2PD96 | Humanin-like 1 | Plays a role as a neuroprotective and antiapoptotic factor. | negative regulation of execution phase of apoptosis |  |  |  |
| MTRNR2L1 | X3F781 | Humanin-like 1 | Plays a role as a neuroprotective and antiapoptotic factor. | negative regulation of execution phase of apoptosis |  |  |  |
| MTRNR2L12 | A0A023KWQ1 | Humanin-like 12 | Plays a role as a neuroprotective and antiapoptotic factor. | negative regulation of execution phase of apoptosis |  |  |  |
| MTRNR2L12 | E1IVY0 | Humanin-like 12 | Plays a role as a neuroprotective and antiapoptotic factor. | negative regulation of execution phase of apoptosis | HMPREF9348_04367 | Uncharacterized protein |  |
| MTRNR2L12 | E9WNY3 | Humanin-like 12 | Plays a role as a neuroprotective and antiapoptotic factor. | negative regulation of execution phase of apoptosis |  |  |  |
| MTRNR2L12 | F8XKK7 | Humanin-like 12 | Plays a role as a neuroprotective and antiapoptotic factor. | negative regulation of execution phase of apoptosis |  |  |  |
| MTRNR2L12 | H4S5L1 | Humanin-like 12 | Plays a role as a neuroprotective and antiapoptotic factor. | negative regulation of execution phase of apoptosis |  |  |  |
| MTRNR2L12 | H4S5N3 | Humanin-like 12 | Plays a role as a neuroprotective and antiapoptotic factor. | negative regulation of execution phase of apoptosis |  |  |  |
| MTRNR2L12 | H5G4G2 | Humanin-like 12 | Plays a role as a neuroprotective and antiapoptotic factor. | negative regulation of execution phase of apoptosis |  |  |  |
| MTRNR2L12 | J7QIY4 | Humanin-like 12 | Plays a role as a neuroprotective and antiapoptotic factor. | negative regulation of execution phase of apoptosis | BN17_38010 | Uncharacterized protein |  |
| MTRNR2L12 | L1FQI3 | Humanin-like 12 | Plays a role as a neuroprotective and antiapoptotic factor. | negative regulation of execution phase of apoptosis |  |  |  |
| MTRNR2L12 | L2CS94 | Humanin-like 12 | Plays a role as a neuroprotective and antiapoptotic factor. | negative regulation of execution phase of apoptosis |  |  |  |
| MTRNR2L12 | R9VPP3 | Humanin-like 12 | Plays a role as a neuroprotective and antiapoptotic factor. | negative regulation of execution phase of apoptosis | H650_16415 | Uncharacterized protein |  |
| MTRNR2L12 | S1L310 | Humanin-like 12 | Plays a role as a neuroprotective and antiapoptotic factor. | negative regulation of execution phase of apoptosis |  |  |  |
| MTRNR2L12 | T6RN53 | Humanin-like 12 | Plays a role as a neuroprotective and antiapoptotic factor. | negative regulation of execution phase of apoptosis |  |  |  |
| MTRNR2L12 | U0HCA7 | Humanin-like 12 | Plays a role as a neuroprotective and antiapoptotic factor. | negative regulation of execution phase of apoptosis |  |  |  |
| MTRNR2L12 | V2RYL8 | Humanin-like 12 | Plays a role as a neuroprotective and antiapoptotic factor. | negative regulation of execution phase of apoptosis |  |  |  |
| MTRNR2L12 | V8TC87 | Humanin-like 12 | Plays a role as a neuroprotective and antiapoptotic factor. | negative regulation of execution phase of apoptosis |  |  |  |
| MTRNR2L12 | X2PD96 | Humanin-like 12 | Plays a role as a neuroprotective and antiapoptotic factor. | negative regulation of execution phase of apoptosis |  |  |  |
| MTRNR2L12 | X3F781 | Humanin-like 12 | Plays a role as a neuroprotective and antiapoptotic factor. | negative regulation of execution phase of apoptosis |  |  |  |
